# Functional roles of ST8SIA3-mediated sialylation of striatal dopamine D_2_ and adenosine A_2A_ receptors

**DOI:** 10.1038/s41398-019-0529-z

**Published:** 2019-08-27

**Authors:** Chien-Yu Lin, Hsing-Lin Lai, Hui-Mei Chen, Jian-Jing Siew, Cheng-Te Hsiao, Hua-Chien Chang, Kuo-Shiang Liao, Shih-Chieh Tsai, Chung-Yi Wu, Ken Kitajima, Chihiro Sato, Kay-Hooi Khoo, Yijuang Chern

**Affiliations:** 1 Institute of Biomedical Sciences, Academia Sinica, Taipei, Taiwan; 2 Taiwan International Graduate Program in Molecular Medicine, National Yang-Ming University and Academia Sinica, Taipei, Taiwan; 3 Institute of Biological Chemistry, Academia Sinica, Taipei, Taiwan; 4 Genomics Research Center, Academia Sinica, Taipei, Taiwan; 5 Department of Research and Development, National Laboratory Animal Center, National Applied Research Laboratories, Taipei and Tainan, Taipei, Taiwan; 6 Bioscience and Biotechnology Center, Nagoya University, Nagoya, 464-860 Japan

**Keywords:** Molecular neuroscience, Pharmacogenetics

## Abstract

Sialic acids are typically added to the end of glycoconjugates by sialyltransferases. Among the six ST8 α-*N*-acetyl-neuraminide α-2,8-sialyltransferases (ST8SIA) existing in adult brains, ST8SIA2 is a schizophrenia-associated gene. However, the in vivo substrates and physiological functions of most sialyltransferases are currently unknown. The ST8SIA3 is enriched in the striatum. Here, we showed that ablation of *St8sia3* in mice (*St8sia3*-KO) led to fewer disialylated and trisialylated terminal glycotopes in the striatum of *St8sia3*-KO mice. Moreover, the apparent sizes of several striatum-enriched G-protein-coupled receptors (GPCRs) (including the adenosine A_2A_ receptor (A_2A_R) and dopamine D_1_/D_2_ receptors (D_1_R and D_2_R)) were smaller in *St8sia3*-KO mice than in WT mice. A sialidase treatment removed the differences in the sizes of these molecules between *St8sia3*-KO and WT mice, confirming the involvement of sialylation. Expression of ST8SIA3 in the striatum of *St8sia3*-KO mice using adeno-associated viruses normalized the sizes of these proteins, demonstrating a direct role of ST8SIA3. The lack of ST8SIA3-mediated sialylation altered the distribution of these proteins in lipid rafts and the interaction between D_2_R and A_2A_R. Locomotor activity assays revealed altered pharmacological responses of *St8sia3*-KO mice to drugs targeting these receptors and verified that a greater population of D_2_R formed heteromers with A_2A_R in the striatum of *St8sia3*-KO mice. Since the A_2A_R-D_2_R heteromer is an important drug target for several basal ganglia diseases (such as schizophrenia and Parkinson’s disease), the present study not only reveals a crucial role for ST8SIA3 in striatal functions but also provides a new drug target for basal ganglia-related diseases.

## Introduction

Glycosylation is effected by the concerted enzymatic action of a series of glycosyltransferases, often in a protein site-specific manner with significant functional impact^1^. Sialic acids are the abundant monosaccharide located on mammalian cell surface glycoconjugates. Sialylation is arguably the most important form of terminal glycosylation capping the glycan chains on glycoproteins and glycolipids performed by sialytransferases (STs)^2^. While single terminal α2,3- or α2,6-sialylation is ubiquitous and has been functionally implicated in a wide range of biological processes, its further extension by the addition of sialic acids via the α2,8-linkage to make di-, tri-, oligo-, or polysialic acid chains is particularly prominent in the brain^3^. A lack of proper sialoglycans in the brain leads to the abnormal development, maintenance, and health of the nervous system^4^. The ST8 α-*N*-acetyl-neuraminide α-2,8-sialyltransferase (ST8Sia) family catalyzes the synthesis of one or multiple α2,8-linked sialic acid chains according to their acceptor specificity^5^. At least five ST8Sia enzymes (ST8SIA1, 2, 3, 4, and 6) have previously been identified in adult brains^6^. These levels of these ST8Sia transferases are developmentally regulated in the brain, suggesting the importance of these enzymes in the nervous system^7^.

ST8SIA2 and 4 are relatively well-characterized particularly for their substrate specificities towards the neural cell adhesion molecule (NCAM), an important regulator of neuronal plasticity in the embryonic brain tissue, and a handful of other acceptor proteins^8^. An *St8sia2* deficiency impairs hippocampal axonal targeting^9^. Genetic studies have associated ST8SIA2 with schizophrenia^10–12^. Conversely, the deletion of *St8sia4* affects polysialic acids (PSA) levels and plasticity at the Schaffer collateral-CA1 synapses^13^. In addition, ST8SIA1 and ST8SIA5 are known to be primarily responsible for mono α2,8-sialylation of GD3, GM1b, GD1a, and GT1b gangliosides^14,15^. ST8SIA3 shares 26% sequence identity with ST8SIA2 and 4. All of these enzymes are considered to be oligo- and polysialyltransferases, but very little is known about the sialylation profiles mediated by ST8SIA3 in vivo.

The mouse *St8sia3* gene shares 96% amino acid identity with human *ST8SIA3*, and is mainly expressed in the brain and testis^16–18^. According to in vitro analyses, ST8SIA3 transfers PSA to NCAM with a lower efficiency than ST8SIA2 and ST8SIA4^4,8^. The apo- and ligand-bound crystal structures of human ST8SIA3 was reported at 1.85-Å resolution and revealed a group of polysiayltransferase-specific structural motifs^19^. ST8SIA3 was also suggested to be the principal sialytransferase responsible for synthesis of the α2,8-trisialic acid (α2,8-triSia) units on gangliosides and glycoproteins in the developing mouse brain because its expression correlated with the α2,8-triSia epitope^7^. To date, only *St8sia2*-knockout (KO) and *St8sia4*-KO, but not *St8sia3*-KO, mice have been generated to determine the biological roles of these enzymes in vivo. The biochemical and pathophysiological properties of ST8SIA3 remain largely unknown.

Because the striatum plays a critical role in coordinating movement and many drug targets (e.g., adenosine A_2A_ receptors, A_2A_R; dopamine D_2_ receptors, D_2_R; and dopamine D_1_ receptors, D_1_R) are enriched in the striatum, we set out to investigate whether the sialylation mediated by ST8SIA3 is essential for the functions of striatum. In the present study, we generated and characterized a mouse model that lacks ST8SIA3. Using this *St8sia3*-KO mouse model and adeno-associated viruses harboring *St8sia3*, we revealed the selective sialylation of several striatum-enriched membrane proteins (including A_2A_R, D_2_R, and D_1_R) by ST8SIA3, which adds α2,8-diSia and α2,8-triSia units to its substrates. The ST8SIA3-mediated sialylation of striatal proteins may affect their distributions in lipid rafts, their abilities to interact with other proteins, and ultimately the motor functions of animals in response to pharmacological modulation of these substrates.

## Materials and methods

### Mice

*St8sia3*-KO mice were generated by CRISPR/Cas9-mediated gene editing as described for details in Supplementary Materials and Methods. Male mice (10–12 weeks old) were analyzed in all experiments except stated otherwise. All animal experimental procedures were performed in accordance with the guidelines established by the Institutional Animal Care and Use Committee (IACUC) at the Institute of Biomedical Sciences, Academia Sinica.

### Immunoblotting

SDS-PAGE and immunoblotting analyses were performed using previously described methods^20^. In brief, protein samples were separated on 10% SDS-PAGE gels and transferred to PVDF membranes (Millipore, Billerica, MA, USA). The membranes were incubated overnight at 4 °C with the primary antibodies listed in Supplementary Table 1, followed by a 1-h incubation with the corresponding secondary antibody conjugated with horseradish peroxidase (Jackson ImmunoResearch Laboratories, West Grove, PA, USA). After extensive washes, immunosignals were detected using the Western Lightning® Plus-ECL Enhanced Chemiluminescence Substrate (PerkinElmer, MA, USA).

### Immunofluorescence staining

Brain sections (20 μm) were subjected to antigen retrieval by boiling in 10 mM citrate buffer (pH 6.0) for 20 min. The sections were subsequently incubated in 3% BSA at room temperature (RT) for 2 h. The sections were incubated with the indicated primary antibody listed in Supplementary Table 1 for 48 h at 4 °C, followed by incubation with the corresponding secondary antibody conjugated with Alexa Fluor 488 or 568 (1:500; Jackson ImmunoResearch Laboratories) in 3% BSA in the dark for 2 h at RT. After extensive washing, nuclei were stained with Hoechst 33342 for 20 min. Images were captured using a Zeiss LSM 780 inverted confocal laser scanning microscope (Axio Observer Z1; Carl Zeiss, Göttingen, Germany) and analyzed using the ZEN 2012 software (Carl Zeiss).

### Glycomic analysis

Glycomic sample preparation of *N*- and *O*-glycans from the striatal membrane fractions were performed exactly as described previously^21^. The permethylated *N*- and *O*-glycans were separately subjected to nanoLC-MS^2^/MS^3^ analysis on an Orbitrap Fusion™ Tribrid™ Mass Spectrometer (Thermo Fisher Scientific, San Jose, CA, USA) interfaced to an UltiMate™ 3000 RSLC nano system (Thermo Fisher Scientific) fitted with a C18 column (Acclaim PepMap® RSLC; Thermo Fisher Scientific).

### Glycan microarray

To fabricating the microarray, series glycans listed in Supplementary Table 2 were prepared and printed onto NHS-coated glass slide (Nexterion H slide; SCHOTT North America, Elmsford, NY, USA) as described previously^22^. S2-566 and A2B5 antibodies were incubated with microarray and the slides were finally scanned with a microarray fluorescence chip reader (GenePix 4300 A; Molecular Devices, Sunnyvale, CA, USA) and scanned images were analyzed with GenePix Pro-6.0 analysis software (Axon Instruments, Foster City, CA, USA).

### Intrastriatal virus injection

The mouse *St8sia3* cDNA was subcloned into an AAV expression vector (pAAV-IRES-hrGFP, Stratagene, La Jolla, CA, USA) driven by the CMV early enhancer/chicken β actin (CAG) promoter and packed into AAV serotype 8 (AAV8) particles as detailed elsewhere^23,24^. Injections were performed at the following stereotaxic coordinates, measured in millimeters (mm) from the bregma: anteroposterior (AP) + 0.5, mediolateral (ML) ± 2.0, and dorsoventral (DV) −2.7 and −3.7. AAV8 particles (AAV-hrGFP or AAV-St8sia3) were injected into the mice of 5 weeks as indicated using a 30-gauge 10-μL Hamilton microsyringe in a volume of 2 μL per intrastriatal site with a titer of 1.0 × 10^10^ vg/μL. The injection rate was 0.5 μL/min and the needle was maintained in place for an additional 5 min after the injection before slow withdrawal of the needle. Eight microliters of viral vectors was injected into four sites in the striatum.

### Preparaton of lipid rafts

Lipid raft fractions were prepared from the striatum using a nondetergent method^25^. In brief, striatal tissues were lysed in lysis buffer (1 mM phenylmethylsulfonyl fluoride, 5 mM NaF, 1% Triton X-100 and 1 mM sodium vanadate in TBS) in the presence of 1x cOmplete™ and EDTA-free protease inhibitor (Roche). The mixture was placed on a shaker for 2 h at 4 °C, and then centrifuged at 800 × *g* for 10 min. Supernatants were further centrifuged at a force of 200 K *g* for 18 h at 4 °C using the SW50.1 rotor (Beckman Coulter) with a discontinuous sucrose gradient (85, 35, and 5% sucrose). Fourteen fractions were separated, and raft fractions were identified with a flotillin-1 antibody by immunoblotting.

### Drug-induced locomotor activity

SCH 58261 (Tocris Bioscience, Bristol, UK) was dissolved in saline containing 15% DMSO and 15% Cremophor EL (Sigma-Aldrich). L-741626 (Tocris Bioscience) was dissolved in a saline solution by adding drops of an acetic acid solution. The pH was adjusted to 7.0 with an NaOH solution. SKF 81297 (Tocris Bioscience) was dissolved in saline. All saline-based solutions also served as the vehicle control. The locomotor activity was examined in a VersaMax activity monitoring system (AccuScan Instruments, Columbus, OH, USA) and quantified for 1 h.

### Statistical analysis

All experiments were reliable and independently conducted at least three times. The sample sizes were similar to other publications. The data in this study meet the assumption of normal distribution and no data was excluded. Materials were collected randomly. Behavior testing and imaging quantitation were performed blinded. Data were analyzed using GraphPad Prism 5 (GraphPad Software, San Diego, CA, USA) software and are presented as the means ± S.E.M. Statistical analysis was performed using either Student’s *t*-test, one-way ANOVA or two-way ANOVA followed by *post hoc* Bonferroni’s multiple comparison tests as indicated. The significant difference was considered when the *P*-value was <0.05. Asterisks were used to indicate degree of significance (*P < 0.05; **P < 0.01; and ***P < 0.001).

## Results

### Expression of ST8SIA3 in the brain

**Fig. 1:**
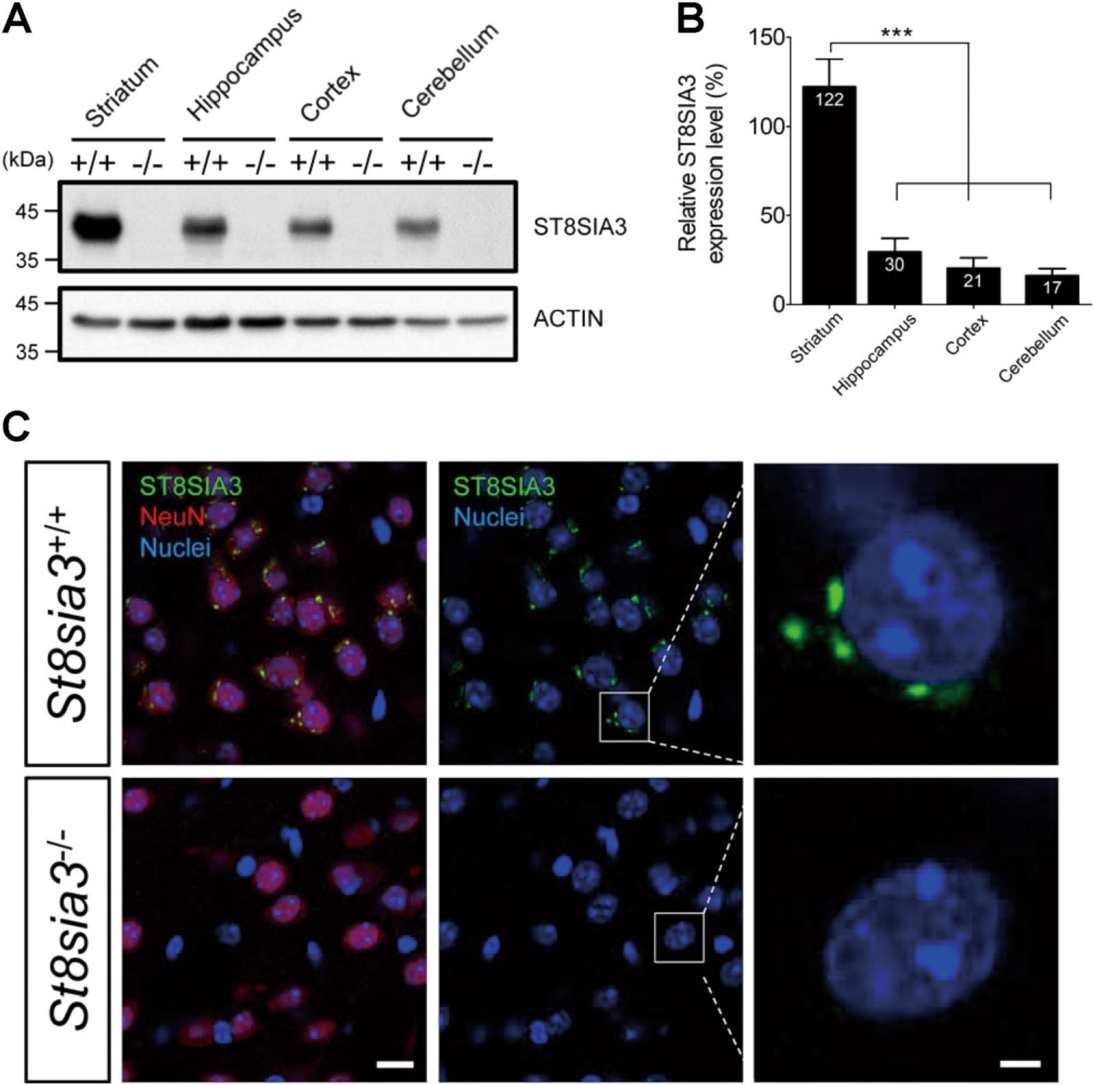
ST8SIA3 is highly expressed in the striatum and is located in NeuN-positive neurons. Brain samples from WT (+/+) and *St8sia3*-KO (−/−) mice were subjected to immunoblotting and immunofluorescence analysis. **a** Immunoblotting of ST8SIA3 from different regions of the mouse brain. The total protein loaded was 30 μg from the striatum, hippocampus, cortex and cerebellum. The data shown are representative of three mice of each genotype. **b** Relative intensity was calculated by using Image J software. *n* *=* 3 for each genotype. ***P < 0.001 (one-way ANOVA followed by Bonferroni’s multiple comparison tests). All values are presented as the means ± S.E.M. **c** Immunofluorescence staining for ST8SIA3 localization (green) and chromatin (Hoechst 33342, blue) in brain sections. Images were captured from striatal regions. The sections were costained with cell-specific markers (red) to help differentiate neurons (NeuN), astrocytes (S100 beta, Supplementary Fig. S1E) and glial cells (Iba1, Supplementary Fig. S1E). ST8SIA3 was detected only in the cytoplasm of NeuN-positive neurons. The data shown are representative of three mice of each genotype. Scale bars, 10 μm (merged panels) and 2 μm (enlarged panels)

*St8sia3*-KO mice were generated by CRISPR/Cas9-mediated gene editing and characterized as described in the Supplementary Results (Supplementary Figs. S1–S3, Supplementary Tables 3 and 4, Supplementary File 1). Immunoblotting analysis showed that ST8SIA3 was expressed in several brain regions of wild-type (WT) mice but not in *St8sia3*-KO mice (Fig. 1a). Moreover, the level of ST8SIA3 was higher in the striatum than in the hippocampus, cortex, and cerebellum (Fig. 1b). Double immunofluorescence staining showed that ST8SIA3 was detected in NeuN-positive neurons (Fig. 1c), but not in S100 beta-positive astrocytes or Iba1-positive glia cells (Supplementary Fig. S1E).

### ST8SIA3 depletion reduced the number of disialyl and trisialyl units on striatal *N*- and *O*-glycans

**Fig. 2:**
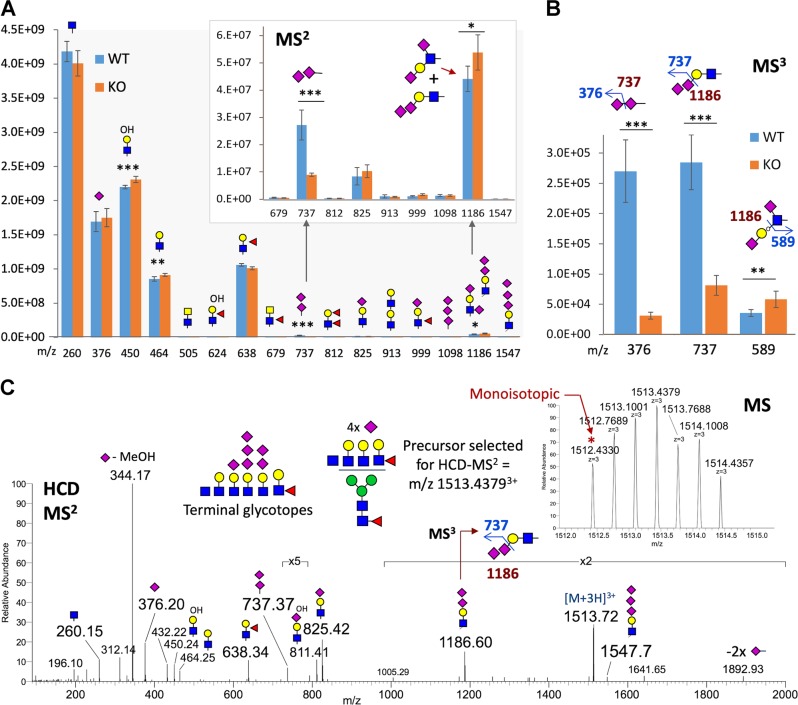
*St8sia3* depletion impaired the addition of terminal sialylated units to striatal *N*-glycans. Permethylated *N*-glycans derived from WT (+/+) and *St8sia3*-KO (−/−) striatal tissues were subjected to a nanoLC-MS^2^-product-dependent MS^3^ analysis, in which the MS^2^ fragment ions representing NeuAc_2_^+^ (*m/z* 737) and NeuAc_2_-Hex-HexNAc^+^ (*m/z* 1186) were additionally selected for MS^3^ analyses upon detection. **a** The summed intensity of a panel of diagnostic MS^2^ ions allowed the rapid assessment of the relative abundance of each glycotope at the glycomic level, as annotated by cartoon symbols and respective *m/z* values indicated on the *x*-axis. The magnified panel showed a significant reduction in the level of the *m/z* 737 ion in *St8sia3*-KO mice. **b** The summed intensity of the diagnostic MS^3^ ions that verified the NeuAc-NeuAc disialyl unit, i.e*., m/z* 376 from 737 and *m/z* 737 from 1186, indicated a drastic reduction in *St8sia3*-KO mice, whereas the ions that identified a type 1 chain sialylated at 2 distinct positions, i.e*., m/z* 589 from 1186, actually increased. The cartoon drawings illustrate how these diagnostic MS^3^ ions validated and added confidence to the identification of the isomeric disialylated glycotopes. **c** A search of the LC-MS^2^/MS^3^ data for presence of terminal trisialylated LacNAc using the diagnostic ions at *m/z* 1547 led to the identification of multisialylated *N*-glycans in the WT samples, but not the *St8sia3*-KO samples, and these antennary glycotopes were observed at low level, along with other permutations of mono- and disialylated antennary glycotopes. The inset shows the correct monoisotopic mass for the selected precursor ion, while the HCD MS^2^ spectrum contained the complement of oxonium ions, supporting the assigned glycosyl composition, including an ion at *m/z* 1186 that was further selected for MS^3^ and yielded a product at *m/z* 737. Glycomic mapping of striatal *N*-glycans revealed a significant reduction in the number of terminal disialyl units in *St8sia3*-KO mice. *n* *=* 3 for each genotype with duplicates. *P < 0.05, **P < 0.01 and ***P < 0.001, as compared with WT control (two-tailed unpaired Student’s *t*-test). All values are presented as the means ± S.E.M

To investigate how ST8SIA3 may impact the overall sialylation of glycoproteins at the glycomic level in the striatum, *N*- and *O*-glycans were sequentially released from the striatal membrane fractions of WT and *St8sia3*-KO mice and subjected to permethylation and nanoLC-MS^2^-pd-MS^3^ analyses. Instead of performing a full systematic analysis of individual *N*-glycan entities, we have resorted to semi-quantitative mapping of the complement of terminal glycotopes collectively presented by the striatal *N*-glycome for the current purpose of determining if any glycotopes were significantly altered in *St8sia3*-KO mice. As shown in our previous study, the summed signal intensity of the diagnostic MS^2^ fragment ions representing a particular terminal glycotope from all MS^2^ spectra^21^. A significant reduction in the total ion intensity of *m/z* 737 corresponding to terminal NeuAc_2_^+^ was observed, whereas the reduction in the intensity of *m/z* 1186 representing a disialylated Gal-GlcNAc was less statistically significant (Fig. 2a). Since both MS^2^ ions were only detected at very low intensity, further MS^3^ was critical not only to ensure true positive results but also to distinguish between the 2 isomeric disialylated glycotopes of *m/z* 1186 (Fig. 2b). Similar summing of the diagnostic MS^3^ ion intensity thus provided a more reliable quantitative index since it was only derived from an MS^2^ precursor ion that displayed a sufficient intensity to yield a productive MS^3^ event, with the disialylated glycotope defined as a pair of MS^2^→MS^3^ transitions, namely, *m/z* 737→376, and *m/z* 1186→737, for authentic NeuAc-NeuAc- and NeuAc-NeuAc-Gal-GlcNAc-, respectively. In this context, we concluded that the reduced levels of these glycotopes in the *St8sia3*-KO striatum were significant, when most other glycotopes, including Lewis X (*m/z* 638) and the isomeric type 1 Gal-3GlcNAc chain disialylated at 2 distinct positions (*m/z* 1186→589), were affected to a lesser or not appreciable extent (Fig. 2b). A few MS^2^ spectra from WT but not *St8sia3*-KO striatal samples contained the ion at *m/z* 1547 for NeuAc_3_Hex_1_HexNAc_1_^+^, albeit at very low intensity (Fig. 2c). Manual verification of one of these spectra against the accurately measured mass of the monoisotopic precursor and its assigned glycosyl composition provided evidence supporting the presence of striatal *N*-glycans carrying a trisialylated LacNAc, among other antennary structures. Although ST8SIA3 was thought to contribute to the synthesis of trisialyl unit^7^, this study presents the first MS data indicating the possible occurrence of this trisialylated LacNAc antennary structure on the *N*-glycans (Fig. 2c). More importantly, the addition of the first NeuAc residue to a monosialylated LacNAc was significantly affected but not completely prevented in the absence of ST8SIA3, which in turn may further impact subsequent oligo- and polysialylation mediated by other ST8SIA enzymes.

Similar nanoLC-MS^2^ analyses applied to the released *O*-glycans also revealed the presence of NeuAc-disialylated and trisialylated simple core 1 *O*-glycans (Supplementary Fig. S4A). Since these smaller *O*-glycans were chromatographically resolved, their relative amounts in WT and *St8sia3*-KO striatal tissues were directly quantified and compared based on their respective peak areas under the extracted ion chromatograms (Supplementary Fig. S4B), and their structures were further verified by MS^2^ analyses (Supplementary Fig. S4C). Overall, a small but significant reduction in the abundance of higher sialylated core 1 *O*-glycans was observed, suggesting that ST8SIA3 may also affect the α2-8-NeuAc-sialylation on *O*-glycans.

**Fig. 3:**
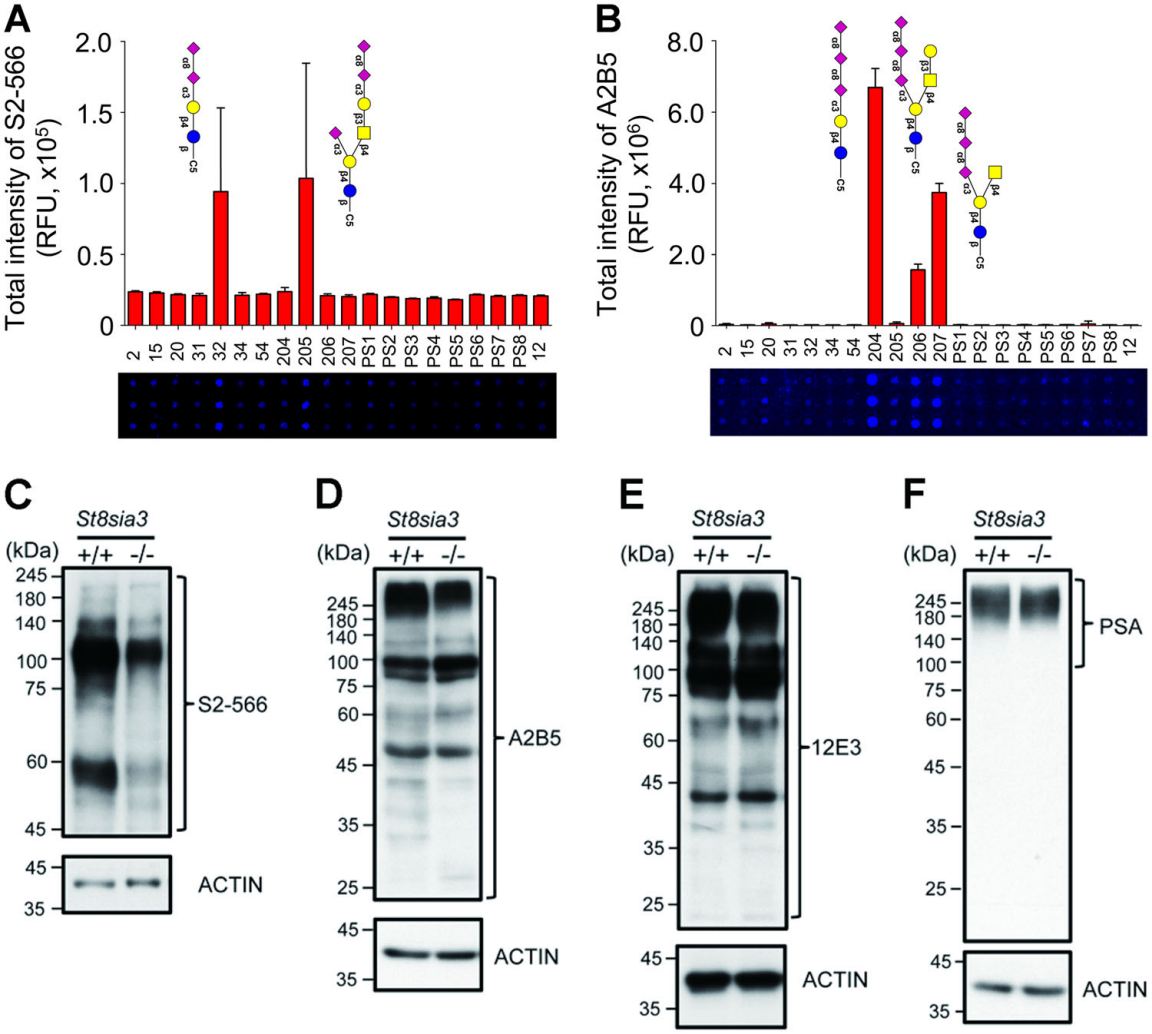
ST8SIA3 deficiency affected the overall sialylation of the striatal glycoproteins. **a**, **b** The quantitative analysis of a glycan microarray for anti-diSia-Gal antibody S2-566 and anti-triSia antibody A2B5 immunoreactivity is shown. Images of the slide obtained from a fluorescence scan after antibody incubation. The grid contains 20 types of glycans listed in Supplementary Table 2. *n* *=* 10 for each glycans with duplicates. **c**, **d** Disialyl and trisialyl units were detected in the striatal lysate. Immunoblotting revealed a significant reduction in the levels of the diSia-Gal and triSia terminal glycotopes in the striatum of *St8sia3*-KO (−/−) mice compared with WT (+/+) control. *n* *=* 3 for each genotype. **e**, **f** Immunoreactivity for the oligoSia and polySia-conjugated glycoproteins was detected using an anti-oligoSia antibody 12E3 and anti-polySia antibody PSA. Similar levels of the oligo-sialylated and poly-sialylated terminal glycotopes were observed in the striatum of WT and *St8sia3*-KO mice. *n* *=* 3 for each genotype. ACTIN was used as an internal loading control

We next evaluated whether the ST8SIA3 deficiency affected the overall sialylation of the striatal glycoproteins in *St8sia3*-KO mice using antibodies that recognize sialic acid residues at the non-reducing terminus^3^. The specificities of the antibodies used in the experiments described below were validated using glycan microarrays^22^. An anti-diSia-Gal antibody, S2-566, which recognizes the Neu5Acα2-8Neu5Acα2-3Gal structures on a glycan microarray (Fig. 3a), detected fewer immunoreactive signals in the immunoblotting of striatal proteins from *St8sia3*-KO mice than in WT mice (Fig. 3c). Similarly, an anti-triSia antibody, A2B5, which recognizes the Neu5Acα2-8Neu5Acα2-8Neu5Ac structures (Fig. 3b), detected lower levels of immunoreactive bands in the striatal proteins from *St8sia3*-KO mice (Fig. 3d), indicating that the striatum of *St8sia3*-KO mice contained fewer di- and trisialylated terminal glycotopes than WT mice. Conversely, the 12E3 antibody that recognizes (Neu5Ac)*n* (where *n* is ≥ 5, Fig. 3e) and an anti-PSA antibody^26^ (where *n* is ≥ 11, Fig. 3f) showed similar expression profiles for the oligo- and polysialic acid-conjugated glycoproteins, respectively, in the striatum from WT and *St8sia3*-KO mice. Consistent with the expression of ST8SIA3 in several brain areas, immunoblotting revealed that compared with those of WT mice, lower levels of diSia- and triSia- terminal glycotopes were detected in the hippocampus, cortex, and cerebellum of *St8sia3-*KO mice (Supplementary Fig. S5). Collectively, these findings suggest that ST8SIA3 mainly synthesizes di- and trisialylated terminal glycotopes in the brain.

### Multiple striatum-enriched proteins are substrates of ST8SIA3

**Fig. 4:**
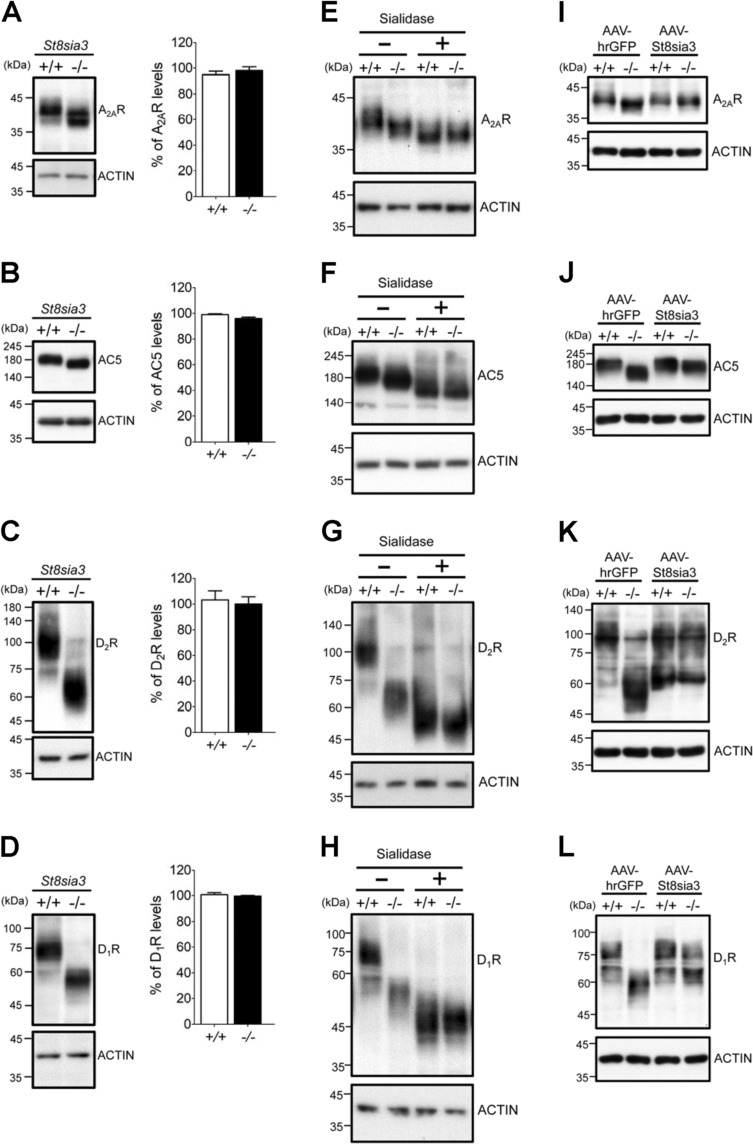
Genetic ablation of *St8sia3* modulated the sialylation patterns of various substrates. **a**–**d** *St8sia3*-KO (−/−) mice showed similar expression levels but larger shifts in the mobility of the major bands for A_2A_R, AC5, D_1_R, and D_2_R in immunoblots of the striatal homogenates compared with WT (+/+). **e**–**h** Differences in the mobilities of these striatum-enriched substrates were eliminated after sialidase treatment. The major bands for A_2A_R, AC5, D_2_R, and D_1_R in striatal samples from both genotypes migrated to lower and similar positions after the enzyme treatment (+) compared with untreated groups (−). **i**–**l** Intrastriatal injections of an AAV virus expressing mouse ST8SIA3 (AAV-St8sia3) or control (AAV-hrGFP). In the *St8sia3*-KO striatum, AAV-St8sia3 rescued the obviously decreased sizes of A_2A_R, AC5, D_2_R, and D_1_R to the original size. *n* *=* 3 for each genotype (two-tailed unpaired Student’s *t*-test). All values are presented as the means ± S.E.M. ACTIN was used as an internal loading control

We next searched for novel protein substrate(s) of ST8SIA3. To date, there is no reported protein sequence or motif for sialylation. We selected nine membrane proteins that contain potential *N*-linked glycosylation sites to assess whether they are substrates of ST8SIA3. Given the enriched expression of ST8SIA3 in the striatum^27^, we first evaluated the apparent sizes of several striatal proteins using immunoblotting analyses. We reasoned that the lack of ST8SIA3 may cause the loss of terminal di- and/or trisialylated glycotopes from its substrates and reduce their apparent molecular weights. At least four of the nine proteins tested showed changes in their apparent molecular weights but no significant change in the total levels of these proteins was observed (Fig. 4). Interestingly, these potential substrates of ST8SIA3, including the adenosine A_2A_ receptor (A_2A_R; Fig. 4a), type V adenylyl cyclase (AC5; Fig. 4b), dopamine D_2_ receptor (D_2_R; Fig. 4c) and dopamine D_1_ receptor (D_1_R; Fig. 4d) are all striatum-enriched proteins^28–30^. Exogenous treatment of striatal proteins with α2-3,6,8-neuraminidase (sialidase; 37 °C for 16 h) removed terminal sialic residues and reduced the apparent molecular weights of all four proteins tested in both WT and *St8sia3*-KO mice (Fig. 4e–h). Most importantly, the differences in the apparent molecular weights of these proteins between WT and *St8sia3*-KO mice disappeared after the treatment with sialidase, suggesting that the ST8SIA3-dependent addition of sialic residues to these striatal proteins may contribute to the changes in the apparent molecular weight between WT and *St8sia3*-KO mice. Because treatment with sialidase further reduced the apparent molecular weights of these striatal proteins in *St8sia3*-KO mice, ST8SIA3 was unlikely to be the only sialyltransferase participating in the sialylation of striatal proteins. No changes in the apparent molecular weights of five glutamate receptors (including NR1, NR2A, NR2B, GluR1, and GluR2) were detected in WT and *St8sia3*-KO mice (Supplementary Fig. S6).

Adeno-associated virus serotype 8 (AAV8) harboring hrGFP or *St8sia3* was intrastriatally injected into 5-week-old *St8sia3*-KO and WT mice to further confirm whether the abnormalities observed in *St8sia3*-KO mice were directly caused by the lack of ST8SIA3 in the striatum. Intrastriatal delivery of AAV8 particles has been shown to effectively express the transgene mainly in neurons and to a much lower level in astrocytes^24^. In addition, under the experimental conditions employed in the present study, intrastriatal delivery of AAV8 particles enabled the transgene to be expressed in most of the striatum, the targeted brain area (Supplementary Fig. S7). At 8 weeks post injection, striatal tissues were harvested and analyzed to determine the apparent molecular weights of A_2A_R, AC5, D_2_R, and D_1_R (Fig. 4i–l); the hrGFP group served as a control. The expression of exogenous ST8SIA3 in the striatum of *St8sia3*-KO mice effectively rescued the differences among the apparent molecular weights of all four proteins tested, supporting the hypothesis that these striatum-enriched G-protein-coupled receptors (GPCRs) and an effector enzyme (AC5) are substrates of ST8SIA3. Consistent with this hypothesis, double immunofluorescence staining also indicated that these striatum-enriched GPCRs (A_2A_R, D_2_R, and D_1_R) are likely to exist in ST8SIA3-expressing striatal neurons (Supplementary Fig. S8).

### The lack of ST8SIA3-mediated modification altered the distributions of ST8SIA3 substrates into lipid rafts

**Fig. 5:**
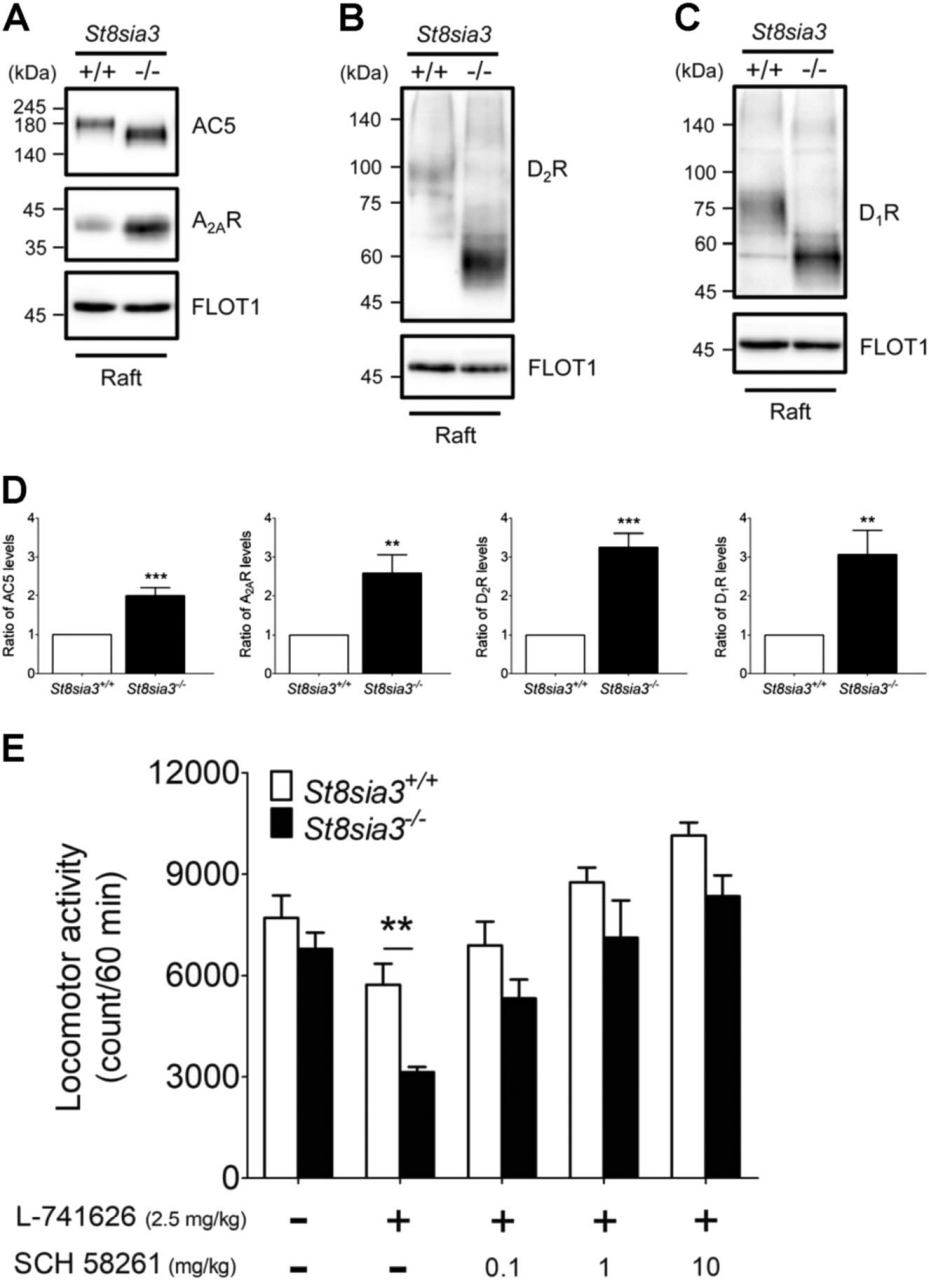
*St8sia3* disruption increased the amount of substrates distributed in lipid rafts and altered the locomotor activities of mice treated with A_2A_R and D_2_R antagonists. **a–c** Lipid raft membrane regions in the striatum of WT (+/+) and *St8sia3*-KO (−/−) mice were isolated using a nondetergent method and analyzed for substrate distribution by immunoblotting. AC5, A_2A_R, D_2_R, and D_1_R were all dispensed in fraction 3 (raft) along with FLOT1, a well-characterized lipid raft marker. **d** Quantitative densitometry analysis was used to measure the intensities of substrates in the lipid raft fractions. ST8SIA3 deficiency significantly increased the amounts of AC5, A_2A_R, D_2_R, and D_1_R in lipid rafts. *n* *=* 15 for each genotype used for seven independent experiments. **P < 0.01 and ***P < 0.001, compared with WT control (two-tailed unpaired Student’s *t*-test). All values are presented as the means ± S.E.M. **e** The A_2A_R antagonist SCH 58261 reversed the effects of the D_2_R antagonist L-741626 on locomotor activity. L-741626 reduced locomotor activity. The difference between WT and *St8sia3*-KO mice was observed at a dose of 2.5 mg/kg. SCH 58261 reversed the differences observed between *St8sia3*-and WT mice at concentrations of 0.1, 1, and 10 mg/kg. *n* *=* 8 for each genotype. **P < 0.01, compared with the WT control (two-way ANOVA followed by Bonferroni’s multiple comparison tests). All values are presented as the means ± S.E.M

To determine the role of ST8SIA3-mediated sialylation in the functions of its substrates, we first evaluated the amounts of these proteins in various biochemical fractions. Immunoblotting revealed that the total levels (Fig. 4a–d), the amounts in the plasma membrane fractions (Supplementary Fig. S9), and the amounts in synaptosome fractions (Supplementary Fig. S10) of A_2A_R, AC5, D_2_R, and D_1_R were similar in the striatum of WT and *St8sia3*-KO mice. We next assessed whether the deficient sialylation of A_2A_R, AC5, D_2_R, and D_1_R affected their abilities to move into lipid rafts, a critical signal transduction microstructure^31^. The lipid raft fractions were isolated from the striatum of WT and *St8sia3*-KO mice using a nondetergent method. The amounts of the target proteins in the raft fraction were analyzed by immunoblotting. FLOT1 is a lipid raft marker^32^. The amounts of ST8SIA3 substrates (A_2A_R, AC5, D_2_R, and D_1_R) in the lipid raft fractions were significantly increased (Fig. 5a–d, Supplementary Fig. S12A–C). No change in the distribution of five glutamate receptors in lipid rafts was observed (Supplementary Figs. S11 and S12D–F). Based on these findings, ST8SIA3-mediated sialylation may affect the distribution of its substrates into lipid rafts, probably changing their functions as well.

### The lack of ST8SIA3-mediated sialylation facilitates the A_2A_R and D_2_R interaction and alters the roles of these proteins in the regulation of motor function in the striatum

Of the four substrates of ST8SIA3, A_2A_R and D_2_R are known to form heteromers, which reciprocally regulate each other in a negative fashion^33^ and have been implicated in movement disorders (e.g., Parkinson’s disease, PD)^34^.

Because the lack of ST8SIA3-mediated sialylation resulted in higher concentrations of A_2A_R and D_2_R in lipid rafts (Fig. 5a–d), we hypothesized that more A_2A_R-D_2_R complexes might exist in lipid rafts. The results of the in situ proximity ligation assay (PLA) using anti-A_2A_R and anti-D_2_R antibodies supported the above hypothesis. More PLA signals were found in the dorsal striatum of *St8sia3*-KO mice than in that of WT mice (Supplementary Fig. S13). Nonetheless, no significant effect on the affinity of ligand binding to A_2A_R and D_2_R (CGS 21680 and sulpride, respectively) or forskolin-evoked AC activity was observed (Supplementary Fig. S14).

Because A_2A_R and D_2_R are known to regulate motor function, we next examined the locomotor activity of *St8sia3*-KO and WT mice. No changes in spontaneous locomotor activity were observed. Treatment with an A_2A_R-selective antagonist (SCH 58261, 1–10 mg/kg, i.p.) dose-dependently increased the locomotor activity of both WT and *St8sia3*-KO mice (Supplementary Fig. S15A). Interestingly, *St8sia3*-KO mice showed a much lower response to SCH 58261 than WT mice. The administration of a D_2_R-selective antagonist (L-741626, 0.25–5 mg/kg, i.p.) reduced the locomotor activity of both WT and *St8sia3*-KO mice in a dose-dependent manner (Supplementary Fig. S15B). In contrast to their inferior response to an A_2A_R antagonist, *St8sia3*-KO mice were more sensitive to L-741626 than WT mice. The differences in the effects of SCH 58261 and L-741626 on locomotor activity between *St8sia3*-KO and WT mice were rescued by restoring ST8SIA3 expression using AAV-St8sia3 (Supplementary Fig. S15D, E), confirming that the altered motor function of *St8sia3*-KO mice depended on ST8SIA3.

Most importantly, A_2A_R antagonist SCH 58261 eliminated the D_2_R inhibitor-induced changes in motor function between WT and *St8sia3*-KO mice (Fig. 5e), suggesting that this altered response to D_2_R blockade in *St8sia3*-KO mice might result from the negative impact of A_2A_R. This observation is consistent with the finding that more A_2A_R form heteromers with D_2_R in the striatum of *St8sia3*-KO mice than in WT mice. Based on these data, ST8SIA3 plays a critical role in the striatum by mediating the sialylation of specific striatal proteins, regulating their distribution in lipid rafts, affecting their interaction with other binding partners, and subsequently modulating striatal functions.

## Discussion

The ST8Sia family is known to catalyze the production of α2,8-linked sialic acid chains according to the acceptor specificity of glycoproteins and gangliosides^5,18,35^. ST8SIA3 has been proposed to deliver α2,8-triSia and polySia units onto glycoproteins in vitro^4,7,8,16^. In the present study, we provided glycomic and immunoblotting evidence that ST8SIA3 mainly mediates the formation of disialylated and trisialylated terminal glycotopes on *N*- and *O*-glycans in the mouse striatum (Figs. 2 and 3a–d, Supplementary Fig. S4). Because the global levels of oligoSia and polySia units remained largely unchanged in mice lacking ST8SIA3 (Fig. 3e, f), in vivo, ST8SIA3 appears to play a role distinct from that of ST8SIA2 and ST8SIA4, enzymes that regulate the adhesive properties of NCAM by polysialylation^8^. Notably, the level of *St8sia4* transcript was slightly decreased in the striatum of *St8sia3-*KO mice (Supplementary Fig. S2D). Whether the level of polySia units on particular substrates of ST8SIA4 was significantly affected would require further investigation. Another interesting observation is that the global di-/trisialylation was not completely lost in *St8sia3*-KO mice (Fig. 3c, d), suggesting that other ST8SIA members may contribute to the di- and trisialylation of glycoproteins. Previous studies suggest that ST8SIA6 may be the other ST8SIA(s) that mediates the di- and trisialylation of glycoproteins, particularly on *O*-linked proteins in the striatum^7,36^. Of note, no alteration in the level of *St8sia6* transcripts was observed in the *St8sia3*-KO striatum (Supplementary Fig. S2F).

At least three GPCRs (i.e., A_2A_R, D_2_R, and D_1_R) and one effector (AC5) were novel sialylated substrates of ST8SIA3 in the striatum (Fig. 4). The extracellular domains of many GPCRs are glycosylated, and glycosylation-mediated regulation varies by receptor type. Specifically, A_2A_R contains one potential *N*-linked glycosylation site^37^, while D_1_R and D_2_R contain two putative sites in their extracellular domains^38,39^. The *N*-glycosylation sites of GPCRs may influence receptor trafficking^40^. Nonetheless, a deficiency in ST8SIA3-mediated sialylation did not affect the total levels or trafficking of A_2A_R, AC5, D_2_R, and D_1_R to either the plasma membrane or synapses in the striatum (Supplementary Figs. S9 and S10). ST8SIA3-mediated sialylation appears to influence the distribution of its substrates in membrane microdomains (such as lipid rafts in Fig. 5). Lipid rafts are enriched in cholesterol and glycosphingolipids and play a critical role in controlling the signaling of GPCRs and other signaling molecules^41^. Depending on the compositions of the available signaling molecules, lipid rafts may regulate signal transduction either positively or negatively^41–43^. Previous studies suggested that D_2_R is largely distributed in the detergent-resistant membrane (DRM) fraction, a restricted microdomain that limits the interaction between D_2_R and other proteins. Biochemical characterization suggests that this DRM fraction does not have the properties of lipid rafts. Conversely, some D_2_Rs located in the detergent-soluble fractions are in a more fluid environment and are allowed to interact with their binding partners^44^. Using a detergent-free method, Vanderwerf et al. demonstrated that D_2_R is highly enriched in the raft fraction^45^. The distribution of A_2A_R in membranes has not yet been extensively characterized. Several studies have shown that A_2A_R colocalizes with TrkB in lipid rafts of motor neurons and regulates the function of TrkB^46,47^. In the present study, we found that removal of diSia/triSia resulted in the enrichment of A_2A_R, D_2_R, and AC5 in lipid rafts, and facilitated their complex formation in lipid rafts. ST8SIA3 may thus significantly contribute to the mechanisms regulating striatal signaling. The reason that removal of terminal diSia and triSia on glycoproteins (at least from A_2A_R, D_2_R, D_1_R, and AC5) would enhance their distribution in lipid rafts remains elusive. It is very likely that removal of di- and triSia would make these proteins less bulky and less negatively charged hence less repulsive. The structure and length of terminal sialic acids may play critical roles in different cellular machineries with specialized functions. Earlier studies indicated that polySia may exist as repulsive polyanionic structures and may mask recognition sites of bioactive molecules^48,49^. The functions of di- and trisialyl glycotopes are largely unknown and require further investigation.

ST8SIA3-mediated sialylation also controls the interactions between its substrates, such as D_2_R and A_2A_R. This observation is of particular interest because these two receptors are known to physically interact and reciprocally regulate each other in a negative fashion in the striatum^50,51^. There are two types of interactions between A_2A_R and D_2_R ligands that target heteromers. Adenosine and A_2A_R ligands decrease the affinity and efficacy of dopamine and D_2_R ligands via allosteric interaction, while dopamine or D_2_R agonists counteract the ability of adenosine or A_2A_R agonists to activate adenylyl cyclase through a canonical Gsα-Giα antagonistic interaction at the adenylyl cyclase level^34,52^. In the striatum, the canonical interaction between A_2A_R and D_2_R ligands depends on the formation of complexes containing A_2A_R-D_2_R heteromers and AC5^53^. Our findings suggest that in the striatum, the major components of this A_2A_R-D_2_R-AC5 complex all undergo ST8SIA3-mediated sialylation in a coordinated manner. Removal of ST8SIA3-dependent sialylation might have facilitated the interaction between A_2A_R and D_2_R, as well as the negative regulation of D_2_R by A_2A_R (Fig. 5c). Since the A_2A_R-D_2_R heteromer is an important drug target for several basal ganglia-related diseases (such as schizophrenia and PD)^52,54^ and because ST8SIA3 is capable of regulating the population of A_2A_R-D_2_R heteromers in the striatum, our findings highlight the importance of ST8SIA3 as a new drug target.

Approximately 34% of Food and Drug Administration-approved drugs target GPCRs^55^. Three of the four ST8SIA3 substrates that we identified are GPCRs (i.e., A_2A_R, D_2_R, and D_1_R). Given the changes in the biochemical properties of these proteins described above, *St8sia3*-KO mice may respond abnormally to agonists or antagonists of these three receptors. In addition to A_2A_R and D_2_R as discussed above, D_1_R is also an important drug target for PD and psychosis^56^. Intake of a selective D_1_R agonist (SKF 81297, 1–10 mg/kg, i.p., Supplementary Fig. S15C) enhanced locomotion in WT mice but not in *St8sia3*-KO mice in the same dose range. The restoration of ST8SIA3 expression in the striatum effectively rescued not only the sialylation of D_1_R (Fig. 4h) but also the abnormal responses of *St8sia3*-KO mice to SKF 81297, which targets D_1_R (Supplementary Fig. S15F). This finding suggests that locomotor-activating responses to D_1_R agonists are dependent on ST8SIA3-mediated sialylation. Interestingly, D_1_R is known to form complexes with the adenosine A_1_ receptor (A_1_R) in the striatum. Similar to the A_2A_R-D_2_R heteromer, A_1_R-D_1_R heteromers are likely to form a signaling complex that contains Gsα, Giα, and AC5^57–59^. Because AC5 is also a substrate of ST8SIA3, ST8SIA3-mediated sialylation may also play a critical role in the regulation of the A_1_R-D_1_R-AC5 complex as observed for the A_2A_R-D_2_R-AC5 complex in the striatum. Further investigation to determine the impact of ST8SIA3-mediated sialylation on drugs targeting the A_1_R-D_1_R-AC5 complex is warranted.

In the brain, sialylation has long been recognized to play an essential role in neuronal development and regeneration^3,4^. The modification of NCAM with PSA is mediated by ST8SIA2 and ST8SIA4 and is associated with development and plasticity in the brain^8,60^. Although ST8SIA3 has been shown to catalyze the polysialylation of NCAM in vitro, it is not as efficient as ST8SIA2 and ST8SIA4^8^. Such modifications with PSA may serve as a negative regulator of cell-cell interactions due to steric repulsion or a binding domain to recruit bioactive molecules such as neurotrophins and dopamine^61,62^. Conversely, much less is known about the functions of di- and trisialyl glycotopes. Using an antibody (i.e., A2B5) that recognizes α2,8-triSia, triSia glycotopes were detected in both developing and adult mouse brains^7^, as shown in Fig. 2d in the present study. Because the expression profile of α2,8-triSia is similar to that of the *St8sia3* gene, ST8SIA3 has been proposed to be the primary ST8SIA family member that synthesizes α2,8-triSia on glycoproteins^7^. Based on the results of the present study, ST8SIA3 is mainly responsible for synthesizing α2,8-triSia on glycoproteins in the striatum. To date, it has not been clearly determined whether the synthesis of PSA by any of the ST8Sia enzymes is accompanied by incompletely elongated oligosialyl chains or whether the shortest chains, namely, the di- and trisialyl units, may be specifically or preferentially added by ST8SIA3 and serve as primers for PSA.

Because ST8SIA3 is enriched in the striatum, it has been implicated in Huntington’s disease (HD), a devastating neurodegenerative disease. Compared to normal controls, the *St8sia3* transcript level is significantly decreased in the caudate of patients with HD and the striatum of HD mice (R6/1)^63^. As shown in the present study, striatal signaling might be altered in patients and mice with HD due to the loss of ST8SIA3 in the striatum. Therefore, patients with HD and normal subjects might respond differently to therapeutic drugs targeting GPCRs that are subject to modification by ST8SIA3. This finding is potentially important because dopamine antagonists have been commonly used to treat chorea and psychosis in patients with HD^64^. Further investigations are required to evaluate whether the alterations in ST8SIA3 activity during HD progression may also alter the pharmacological properties of drugs targeting ST8SIA3 substrates (e.g., D_2_R).

In summary, ST8SIA3 is the principle ST8Sia family member that mediates the synthesis of diSia and triSia on glycoproteins and thus regulates their distribution and mode of action in the striatum. Several ST8SIA3 substrates (e.g., A_2A_R, D_2_R, and D_1_R) have major roles in controlling striatal functions. Our findings provide new insights into the mechanisms regulating the striatum and may pave the way for the development of novel therapeutic strategies for basal ganglia-related diseases.

## Supplementary information

Supplementary Information_clean version

Supplementary File

Supplementary Fig. S1

Supplementary Fig. S2

Supplementary Fig. S3

Supplementary Fig. S4

Supplementary Fig. S5

Supplementary Fig. S6

Supplementary Fig. S7

Supplementary Fig. S8

Supplementary Fig. S9

Supplementary Fig. S10

Supplementary Fig. S11

Supplementary Fig. S12

Supplementary Fig. S13

Supplementary Fig. S14

Supplementary Fig. S15

Supplementary Information_marked-up version

## References

[1] Spiro RG (2002). Protein glycosylation: nature, distribution, enzymatic formation, and disease implications of glycopeptide bonds. Glycobiology.

[2] Rao FV (2009). Structural insight into mammalian sialyltransferases. Nat. Struct. Mol. Biol..

[3] Sato C, Kitajima K (2013). Disialic, oligosialic and polysialic acids: distribution, functions and related disease. J. Biochem..

[4] Schnaar RL, Gerardy-Schahn R, Hildebrandt H (2014). Sialic acids in the brain: gangliosides and polysialic acid in nervous system development, stability, disease, and regeneration. Physiol. Rev..

[5] Paulson JC, Rademacher C (2009). Glycan terminator. Nat. Struct. Mol. Biol..

[6] Harduin-Lepers A (2001). The human sialyltransferase family. Biochimie.

[7] Inoko E (2010). Developmental stage-dependent expression of analpha2,8-trisialic acid unit on glycoproteins in mouse brain. Glycobiology.

[8] Angata K (2000). Differential biosynthesis of polysialic acid on neural cell adhesion molecule (NCAM) and oligosaccharide acceptors by three distinct alpha 2,8-sialyltransferases, ST8Sia IV (PST), ST8Sia II (STX), and ST8Sia III. J. Biol. Chem..

[9] Angata K (2004). Sialyltransferase ST8Sia-II assembles a subset of polysialic acid that directs hippocampal axonal targeting and promotes fear behavior. J. Biol. Chem..

[10] Arai M (2006). Association between polymorphisms in the promoter region of the sialyltransferase 8B (SIAT8B) gene and schizophrenia. Biol. Psychiatry.

[11] Yang SY (2015). Association between ST8SIA2 and the risk of schizophrenia and bipolar I disorder across diagnostic boundaries. PLoS ONE.

[12] Kamien B (2014). Characterization of a 520 kb deletion on chromosome 15q26.1 including ST8SIA2 in a patient with behavioral disturbance, autism spectrum disorder, and epilepsy. Am. J. Med. Genet. A.

[13] Eckhardt M (2000). Mice deficient in the polysialyltransferase ST8SiaIV/PST-1 allow discrimination of the roles of neural cell adhesion molecule protein and polysialic acid in neural development and synaptic plasticity. J. Neurosci..

[14] Kim YJ (1997). Molecular cloning and expression of humanalpha2,8-sialyltransferase (hST8Sia V). Biochem. Biophys. Res. Commun..

[15] Sasaki K (1994). Expression cloning of a GM3-specific alpha-2,8-sialyltransferase (GD3 synthase). J. Biol. Chem..

[16] Yoshida Y, Kojima N, Kurosawa N, Hamamoto T, Tsuji S (1995). Molecular cloning of Sia alpha 2,3Gal beta 1,4GlcNAc alpha 2,8-sialyltransferase from mouse brain. J. Biol. Chem..

[17] Yoshida Y (1996). Unique genomic structure and expression of the mouse alpha 2,8-sialyltransferase (ST8Sia III) gene. Glycobiology.

[18] Lee YC (1998). Cloning and expression of cDNA for a human Sia alpha 2,3Gal beta 1, 4GlcNA:alpha 2,8-sialyltransferase (hST8Sia III). Arch. Biochem. Biophys..

[19] Volkers G (2015). Structure of human ST8SiaIII sialyltransferase provides insight into cell-surface polysialylation. Nat. Struct. Mol. Biol..

[20] Chien T (2018). GSK3beta negatively regulates TRAX, a scaffold protein implicated in mental disorders, for NHEJ-mediated DNA repair in neurons. Mol. Psychiatry.

[21] Hsiao CT (2017). Advancing a high throughput glycotope-centric glycomics workflow based on nanoLC-MS(2)-product dependent-MS(3) analysis of permethylated glycans. Mol. Cell. Proteomics.

[22] Wang CC (2008). Glycan microarray of Globo H and related structures for quantitative analysis of breast cancer. Proc. Natl Acad. Sci. USA.

[23] Aschauer DF, Kreuz S, Rumpel S (2013). Analysis of transduction efficiency, tropism and axonal transport of AAV serotypes 1, 2, 5, 6, 8 and 9 in the mouse brain. PLoS ONE.

[24] Pignataro D (2017). Adeno-associated viral vectors serotype 8 for cell-specific delivery of therapeutic genes in the central nervous system. Front. Neuroanat..

[25] Persaud-Sawin DA, Lightcap S, Harry GJ (2009). Isolation of rafts from mouse brain tissue by a detergent-free method. J. Lipid Res..

[26] Sato C (1995). Characterization of the antigenic specificity of four different anti-(alpha 2–>8-linked polysialic acid) antibodies using lipid-conjugated oligo/polysialic acids. J. Biol. Chem..

[27] Mazarei G (2010). Expression analysis of novel striatal-enriched genes in Huntington disease. Hum. Mol. Genet..

[28] Schiffmann SN, Jacobs O, Vanderhaeghen JJ (1991). Striatal restricted adenosine A2 receptor (RDC8) is expressed by enkephalin but not by substance P neurons: an in situ hybridization histochemistry study. J. Neurochem..

[29] Levey AI (1993). Localization of D1 and D2 dopamine receptors in brain with subtype-specific antibodies. Proc. Natl Acad. Sci. USA.

[30] Matsuoka I, Suzuki Y, Defer N, Nakanishi H, Hanoune J (1997). Differential expression of type I, II, and V adenylyl cyclase gene in the postnatal developing rat brain. J. Neurochem..

[31] Simons K, Ehehalt R (2002). Cholesterol, lipid rafts, and disease. J. Clin. Invest.

[32] Babuke T, Tikkanen R (2007). Dissecting the molecular function of reggie/flotillin proteins. Eur. J. Cell Biol..

[33] Ferre S (2011). Adenosine A(2A) receptors and A(2A) receptor heteromers as key players in striatal function. Front. Neuroanat..

[34] Taura J (2018). Behavioral control by striatal adenosine A2A -dopamine D2 receptor heteromers. Genes Brain Behav..

[35] Harduin-Lepers A (2008). Evolutionary history of thealpha2,8-sialyltransferase (ST8Sia) gene family: tandem duplications in early deuterostomes explain most of the diversity found in the vertebrate ST8Sia genes. BMC Evol. Biol..

[36] Takashima S (2002). Molecular cloning and expression of a sixth type of alpha 2,8-sialyltransferase (ST8Sia VI) that sialylates O-glycans. J. Biol. Chem..

[37] Piirainen H, Ashok Y, Nanekar RT, Jaakola VP (2011). Structural features of adenosine receptors: from crystal to function. Biochim. Biophys. Acta.

[38] Min C (2015). N-linked glycosylation on the N-terminus of the dopamine D2 and D3 receptors determines receptor association with specific microdomains in the plasma membrane. Biochim. Biophys. Acta.

[39] Karpa KD, Lidow MS, Pickering MT, Levenson R, Bergson C (1999). N-linked glycosylation is required for plasma membrane localization of D5, but not D1, dopamine receptors in transfected mammalian cells. Mol. Pharmacol..

[40] Schoneberg T, Schulz A, Gudermann T (2002). The structural basis of G-protein-coupled receptor function and dysfunction in human diseases. Rev. Physiol. Biochem. Pharmacol..

[41] Pike LJ (2003). Lipid rafts: bringing order to chaos. J. Lipid Res..

[42] Zhu X (2008). Increased cellular free cholesterol in macrophage-specific Abca1 knock-out mice enhances pro-inflammatory response of macrophages. J. Biol. Chem..

[43] Triantafilou M, Miyake K, Golenbock DT, Triantafilou K (2002). Mediators of innate immune recognition of bacteria concentrate in lipid rafts and facilitate lipopolysaccharide-induced cell activation. J. Cell Sci..

[44] Sharma M, Celver J, Octeau JC, Kovoor A (2013). Plasma membrane compartmentalization of D2 dopamine receptors. J. Biol. Chem..

[45] Vanderwerf SM (2015). Role for Rab10 in methamphetamine-induced behavior. PLoS ONE.

[46] Sebastiao AM, Assaife-Lopes N, Diogenes MJ, Vaz SH, Ribeiro JA (2011). Modulation of brain-derived neurotrophic factor (BDNF) actions in the nervous system by adenosine A(2A) receptors and the role of lipid rafts. Biochim. Biophys. Acta.

[47] Mojsilovic-Petrovic J (2006). Protecting motor neurons from toxic insult by antagonism of adenosine A2a and Trk receptors. J. Neurosci..

[48] Varki A (1993). Biological roles of oligosaccharides: all of the theories are correct. Glycobiology.

[49] Hildebrandt H, Becker C, Murau M, Gerardy-Schahn R, Rahmann H (1998). Heterogeneous expression of the polysialyltransferases ST8Sia II and ST8Sia IV during postnatal rat brain development. J. Neurochem..

[50] Ferre S, Fuxe K, von Euler G, Johansson B, Fredholm BB (1992). Adenosine-dopamine interactions in the brain. Neuroscience.

[51] Chen JF (2001). The role of the D(2) dopamine receptor (D(2)R) in A(2A) adenosine receptor (A(2A)R)-mediated behavioral and cellular responses as revealed by A(2A) and D(2) receptor knockout mice. Proc. Natl Acad. Sci. USA.

[52] Ferre S (2016). Allosteric mechanisms within the adenosine A2A-dopamine D2 receptor heterotetramer. Neuropharmacology.

[53] Navarro G (2018). Evidence for functional pre-coupled complexes of receptor heteromers and adenylyl cyclase. Nat. Commun..

[54] Beaulieu JM, Gainetdinov RR, Caron MG (2007). The Akt-GSK-3 signaling cascade in the actions of dopamine. Trends Pharm. Sci..

[55] Hauser AS (2018). Pharmacogenomics of GPCR drug targets. Cell.

[56] Cadet JL, Jayanthi S, McCoy MT, Beauvais G, Cai NS (2010). Dopamine D1 receptors, regulation of gene expression in the brain, and neurodegeneration. CNS Neurol. Disord. Drug Targets.

[57] Ferre S, Diaz-Rios M, Salamone JD, Prediger RD (2018). New developments on the adenosine mechanisms of the central effects of caffeine and their implications for neuropsychiatric disorders. J. Caffeine Adenosine Res..

[58] Ferre S (2008). An update on the mechanisms of the psychostimulant effects of caffeine. J. Neurochem..

[59] Ciruela F (2006). Presynaptic control of striatal glutamatergic neurotransmission by adenosine A1-A2A receptor heteromers. J. Neurosci..

[60] Hildebrandt H, Muhlenhoff M, Weinhold B, Gerardy-Schahn R (2007). Dissecting polysialic acid and NCAM functions in brain development. J. Neurochem..

[61] Sato C, Hane M, Kitajima K (2016). Relationship between ST8SIA2, polysialic acid and its binding molecules, and psychiatric disorders. Biochim. Biophys. Acta.

[62] Kanato Y, Kitajima K, Sato C (2008). Direct binding of polysialic acid to a brain-derived neurotrophic factor depends on the degree of polymerization. Glycobiology.

[63] Desplats PA (2007). Glycolipid and ganglioside metabolism imbalances in Huntington’s disease. Neurobiol. Dis..

[64] Frank S (2014). Treatment of Huntington’s disease. Neurotherapeutics.

