# Supplementary material for: Functional roles of ST8SIA3-mediated sialylation of striatal dopamine D2 and adenosine A2A receptors
Source: Transl Psychiatry. 2019 Aug 27;9:209. doi: 10.1038/s41398-019-0529-z (PMC6712005; doi:10.1038/s41398-019-0529-z)
Supplement: Supplementary file 1 — Supplementary Information_clean version [file 41398_2019_529_MOESM1_ESM.pdf]

SUPPLEMENTARY INFORMATION for

Functional roles of ST8SIA3-mediated sialylation of striatal dopamine D_2 and adenosine A_{2A} receptors

Running Title

ST8SIA3 regulates $A_{2A}R$ and D_2R heteromerization

Authors

Chien-Yu Lin, Hsing-Lin Lai, Hui-Mei Chen, Jian-Jing Siew, Cheng-Te Hsiao, Hua-Chien Chang, Kuo-Shiang Liao, Shih-Chieh Tsai, Chung-Yi Wu, Ken Kitajima, Chihiro Sato, Kay-Hooi Khoo, Yijuang Chern*

***Correspondence and requests for materials should be addressed to:**

Yijuang Chern, Ph.D., Institute of Biomedical Sciences, Academia Sinica, Taipei, Taiwan. Email: bmychern@ibms.sinica.edu.tw

Supplementary Materials and Methods

Supplementary Results

Supplementary Tables

Supplementary File

Supplementary Reference

Supplementary Figure Legends

Supplementary Figures

Supplementary Materials and Methods

DNA Construction and *In Vitro* Transcription.

The murine *St8sia3* exons 3-4, including the start codon, were selected as targeting regions to prepare *St8sia3*-targeted alleles using the CRISPR/Cas9 nuclease system[1]. Two sgRNA targeting sequences (sgRNA targets exon 3: TGGAGATGGCAACCTCGC and sgRNA targets exon 4-intron4/5: TTTTACATCAAAGGTAGGAT) were designed and showed very high specificity for murine *St8sia3*. *St8sia3* sgRNA oligonucleotides were annealed and cloned into the pUC57-sgRNA expression vector (plasmid #51132; Addgene). The sgRNA expression vectors were linearized by *Hind*III digestion and used as DNA templates for *in vitro* transcription with the MEGAshortscript Kit (#AM1354; Ambion). Transcribed sgRNAs were purified using the MEGAclear Kit (#AM1908; Ambion) and concentrated by alcohol precipitation. For Cas9 mRNA production, the pST1374-Cas9-N-NLS-flag-linker vector (plasmid #44758; Addgene) was linearized with *Xma*I and *Mfe*I digestion and subsequently transcribed *in vitro* using the T7 mMESSAGE mMACHINE transcription kit (#AM1344; Ambion). The Cas9-N-NLS-flag-linker mRNA was purified using the MEGAclear Kit (Ambion). The concentrations of sgRNAs and the Cas9 mRNA were measured and RNA quality was evaluated using an agarose gel.

CRISPR/Cas9 RNA Pronuclear Microinjection and Mutant Analysis.

Mouse zygotes were obtained by mating males with superovulated C57BL/6JNarl females. Two sgRNAs (50 ng of each) and Cas9 (20 ng) mRNA mixtures were prepared in RNase-free water and microinjected into the male pronucleus of zygotes. These two sgRNAs and the Cas9 protein induce DNA-double strand breaks across the *St8sia3* targeting sites. Non-homologous end joining (NHEJ) directly rejoined the two broken ends and led to a deletion of the DNA

fragment. The blastocysts derived from the injected zygotes were implanted into pseudopregnant female mice, where they developed into a mouse containing the altered *St8sia3* gene. Genomic DNA samples were obtained from tail biopsies of founder mice and subjected to PCR of the CRISPR/Cas9 targeting site with the primer pair: AGCTGGTGTTTAGCCTCCAC and TGGTGTTCAGTCAAGGCCA. PCR amplicons with the DNA deletion and insertion were cloned into pGEM-T vector (Promega) for the sequencing analysis.

Mice Breeding and Genotyping.

All *St8sia3*-KO and WT mice were bred and housed in a credited specific-pathogen-free (SPF) facility on a 12-h light/dark cycle, supplied with food and water *ad libitum* and provided standard care according to laboratory animal care policies. Germline transmission of the *St8sia3*-targeted allele was achieved by breeding the founders to C57BL/6JNarl strains. Heterozygous offspring (*St8sia3*$^{+/-}$) were intercrossed to generate homozygous pups (*St8sia3*$^{-/-}$). Genomic DNA was isolated from mouse tail biopsies using the Wizard® Genomic DNA Purification Kit (Promega). Genotyping of successive progeny was conducted by PCR of the tail DNA using the oligonucleotide primers FP plus RP1 for wild-type alleles and FP plus RP2 for targeted alleles (FP: 5'-CCCTCCCCTTGGTTATTTA-3'; RP1: 5'-CCTAGCACACTGGCGACTC-3'; RP2: 5'-TGGCTACGGATCATCTCCTCTT-3'). The amplification reaction conditions were: 95°C for 3 min, followed by 35 cycles at 95°C for 30 sec, 58°C for 30 sec, and 72°C for 30 sec, followed by a final extension step at 72°C for 7 min. The PCR product sizes were 515 bp (wild-type), 312 bp (targeted, #16), 295 bp (targeted, #49) and 301 bp (targeted, #59) on 3% Etbr-stained agarose gels.

RNA Analysis.

RT-PCR was used to detect the levels of the mRNAs encoding *St8sia3* and other members of the *St8sia* family (1, 2, 4, 5 and 6). Total RNA was extracted from the mouse striatum using a GENEzol TriRNA Pure Kit (Geneaid), according to the manufacturer's instructions. The RNA was eluted with diethylpyrocarbonate (DEPC)-treated water. First-strand cDNAs were synthesized using SuperScript™ III Reverse Transcriptase (Invitrogen) with random hexamers. All *St8sia* transcripts were amplified using specific sets of primers listed in Supplementary Table 5. Simultaneously, *Gapdh* primers were used for quality and quantity control of cDNA preparations.

Protein Extraction and Quantification.

To purify total lysates, brain tissues were obtained, homogenized and lysed in radioimmunoprecipitation assay buffer (RIPA; 150 mM sodium chloride, 1.0% Triton X-100, 0.5% sodium deoxycholate, 0.1% SDS and 50 mM Tris, pH 8.0) in the presence of 1% v/v protease inhibitor cocktail (Sigma-Aldrich). The mixture was placed on ice for 30 minutes, and then centrifuged at 40,000 × *g* for 1 hour at 4°C.

To purify plasma membrane fractions, brain tissues were lysed in homogenization buffer (1 mM EGTA, 1 mM $MgCl_2$, 10 nM okadaic acid, 100 μM, phenylmethylsulfonyl fluoride, 40 μM leupeptin, 25 mM Tris-HCl buffer, pH8.0) containing 1% v/v protease inhibitor cocktail (Sigma-Aldrich). The homogenate was first centrifuged at 500 × *g* for 10 minutes at 4°C to remove debris. The supernatant was collected and centrifuged at 50,000 × *g* for 1 hour at 4°C to collect membrane fraction in the pellets Pellets were suspended with in an ice-cold lysis buffer (0.2 mM EGTA, 0.2 mM $MgCl_2$, 30 nM okadaic acid, 40 μM phenylmethylsulfonyl fluoride, 0.1

mM leupeptin, 0.2 mM sodium orthovanadate, and 20 mM HEPES, pH 8.0 plus 1X protease inhibitor mixture). The protein concentration of each sample was measured by the Bradford method (Bio-Rad) using serial dilutions of BSA as the standards.

To prepare the synaptic plasma membrane fractions, a discontinuous sucrose gradient analysis was performed using previously described methods[2]. Briefly, brain tissues were lysed in 0.32 M HEPES-buffered sucrose solution. The homogenate was first centrifuged at 800 × *g* for 10 minutes at 4°C to remove the nuclear fraction. The supernatant was centrifuged at 10,000 × *g* for 15 minutes at 4°C to separate the cytosolic fraction and crude synaptosomal fraction existing in the pellets. Pellets were lysed by hypoosmotic shock in ddH_2O, rotated in 4 mM HEPES at 4°C for 30 minutes and centrifuged at 25,000 × *g* for 20 minutes to separate the crude vesicular fraction supernatant and the synaptosomal membrane fraction pellets. Fractionation of the synaptic plasma membrane was performed by centrifuging in a swinging bucket rotor at 150,000 × *g* for 2 hours at 4°C in 0.8M / 1.0 M / 1.2 M HEPES-buffered sucrose solution. The synaptic plasma membrane fraction was further centrifuged in a fixed angle rotor at 200,000 × *g* for 30 minutes at 4°C. Resuspend the synaptic plasma membrane pellet in the 50 mM HEPES / 2 mM EDTA solution and the protein concentration of each sample was measured by the BCA Protein Assay Kit (Thermo Fisher Scientific).

Mating Test

To assess embryonic viability and sterile phenotypes of *St8sia3*-KO mice, controlled breeding experiments were conducted, and the number of pups sired recorded. Male and female mice at their eightieth postnatal day (P80) were defined totally sexual maturation and intercrossed. The total numbers of litters and pups were recorded for each pair, and the average

litter sizes were analyzed.

Grip Strength

The forelimb grip strength of mice was measured by MK-380CM/R Grip Strength meter (Muromachi Kikai Co.). For this measurement, the mouse was performed the grip capability or muscular power for five trials with 10-min intervals.

Hot Plate Test

This test is used to measure the thermal nociception of mice and the standard setting temperature is set at 55°C. The tested mouse is put on the surface of a metal plate which provides heat stimulation (Hot/Cold Plate 35100 model; Ugo Basile). The degree of nociceptive threshold can be measured via the withdrawal latency when the tested mouse shows sign of lifting or licking hind paws or jumping.

Tail-flick Test

The tail-flick test is designed to assess nociceptive threshold by using infrared source (Tail Flick Unit 37360 model; Ugo Basile). Radiant heat is generated from an I. R. source (50W bulb) with adjustable energy level. Ugo Basile Heat Flux I.R. Radiometer 37300 is an instrument for I.R. source adjustment. The tested mouse is anesthesia free and is covered with a linen glove with its tail sitting on the groove of the heating panel. Wait until the mouse calms down then introduce the infrared radiant heat onto the subject and record the latency of tail flick.

Electronic von Frey Test

The von Frey test is used to assess mechanical nociception by IITC Electronic von Frey Anesthesiometer 2392. A non-anesthesia mouse is placed in a small acrylic box on a confined area on wire mesh to limit its action. When the tested mouse calms down, use a special Rigid Tip (Rigid Tip+ 0.01 inches tungsten electrode) to poke its hind paw to measure the mechanical threshold which may induce withdrawal response.

Rotarod Test.

The rotarod test was designed to evaluate the motor coordination and balance of the mice by forcing the subject to run on the rotarod (#7650 Accelerating model; Ugo Basile). Mice were placed on the rotating rod at an acceleration speed of 12-40 rpm (4 rpm increment every 30 seconds). The duration that each animal remained on the rotarod was recorded for up to a maximum of 5 minutes. Each mouse received three daily trials with 15 minute-intervals.

Modified-SHIRPA

Modified SHIRPA is a comprehensive, standardized, and high-throughput protocol that is compatible with the original "SHIRPA" protocol[3, 4]. The modified SHIRPA protocol includes 42 tests (33 behavioral observations, 7 metabolic or disease observations and 2 additional comments on behavior in the arena protocol) and 16 new criteria for the morphological appearance that were designed to screen for developmental and metabolic anomalies. Behavioral observations include gait, balance, various reflection assays, and startle responses. All behaviors are scored to provide a semi-quantitative assessment of each parameter. The assessment provides an estimate of the behavioral profile of the animal. It also provides indications about specific behavioral anomalies resulting from muscular, lower motor neuronal, spinocerebellar, sensory and

autonomic defects.

Glycomic Analysis by NanoLC-MS^2/MS^3.

Glycomic sample preparation including the sequential release of *N*- and *O*-glycans from the striatal membrane fractions, permethylation and subsequent clean-up were performed exactly as described previously[5]. The permethylated *N*- and *O*-glycans were separately subjected to nanoLC-MS^2/MS^3 analysis on an Orbitrap Fusion™ Tribrid™ Mass Spectrometer (ThermoFisher Scientific) interfaced to an UltiMate™ 3000 RSLC nano system (ThermoFisher Scientific) fitted with a C18 column (Acclaim PepMap® RSLC; ThermoFisher Scientific). The instrument settings and nanoLC conditions for a data dependent acquisition of MS^2 coupled with a product dependent MS^3 data acquisition (for MS^2 ions at *m/z* 638, 737 and 1186) in a 3-sec Top Speed mode and subsequent data analysis using the GlyPick program have been described in detail in previous work[5]. HCD MS^2 data were mass measured in Orbitrap at 30,000 resolution for a mass accuracy within 5 ppm, whereas the product dependent MS^3 were acquired in the ion trap for highest sensitivity. For comparative analyses between pairs of WT and *St8sia3*-KO sample triplicates acquired consecutively using identical settings on the same instrument, the summed intensities of all selected diagnostic MS^2 ions were used to ensure total sample amount consistency and normalize for the slight run-to-run variations. Select MS^2 and MS^3 spectra relevant to the inferred structures reported in this work were further manually examined and interpreted.

Microarray Fabrication and Detection.

To fabricating the microarray, series glycans listed in Supplementary Table 2 were prepared

by dissolving in the printing buffer (300 mM phosphate buffer and 0.005% Tween 20, pH 8.5) in 100 M concentration[6]. Microarrays were printed (BioDot; Cartesian Technologies) by robotic pin (SMP3; TeleChem International) deposition of 0.6 nL of various solutions from 96-well plate onto NHS-coated glass slide (Nexterion H slide; SCHOTT North America). The microarray was designed 16 grids in one slide, and 12 columns × 12 rows in one grid. Printed slides were allowed to react in an atmosphere of 80% humidity for an hour followed by desiccation overnight.

Glycan Microarray and Antigen Immunogenicity Comparison.

Before serum antibody binding, the glycan microarrays were blocked with Superblock blocking buffer (Pierce) at 4°C for 1 hour, followed by washing with PBST (PBS containing 0.05% tween 20) buffer twice. The mouse monoclonal antibodies S2-566 and A2B5 were diluted to 5 g/mL with PBST, followed by incubating with microarray separately at 4°C for overnight. Excess antibodies were washed out and the microarrays were incubated with anti-mouse IgG antibody labeled with fluorescence as the secondary antibody at 4°C in dark for 1 hour. The slides were then washed thoroughly and scanned at 488 nm wavelength with a microarray fluorescence chip reader (GenePix 4300A; Molecular Devices Corporation) and scanned images were analyzed with GenePix Pro-6.0 analysis software (Axon Instruments).

Enzymatic Protein Deglycosylation.

Sialidase (neuraminidase) is very well suited for the complete removal of sialic acids from *N*-linked or *O*-linked glycans, which are α2,3-, α2,6-, or α2,8-linked to oligosaccharides and glycoproteins in a wide variety of biological materials. Striatal protein lysates (80 μg) were

treated with 0.01 units of sialidase (Roche) in reaction buffer (5 mM $CaCl_2$ and 50 mM sodium acetate, pH 5.5) and incubated at 37°C for 16 hours.

Construction and Production of Recombinant AAV-8 Vectors.

The mouse *St8sia3* cDNA was synthesized from striatal RNA isolated by TRIzol (Invitrogen) and treated with DNase (Invitrogen) using a SuperScript II Reverse Transcriptase Kit (Invitrogen). The *St8sia3* cDNA was cloned into the TA cloning site of a pcDNA3.1/V5-His-TOPO vector (Invitrogen), amplified, excised and subcloned into the *Xho*I sites of an AAV8 expression vector (pAAV-IRES-hrGFP; Stratagene). This recombinant AAV (rAAV) plasmid is named pAAV-St8sia3-hrGFP (short form: AAV-St8sia3), in which *St8sia3* and a reporter gene, hrGFP, are driven by the CMV early enhancer/chicken β actin (CAG) promoter. The recombinant pAAV-St8sia3-hrGFP vector and the control pAAV-IRES-hrGFP vector (short form: AAV-hrGFP) were constructed, packaged, purified and tittered. Three plasmids (the transfer plasmid, the *trans* rep-cap plasmid, and the packaging helper plasmid) were co-transfected into human embryonic kidney 293 (HEK293) cells to produce infectious AAV-8 particles. The cell lysate was purified via cesium chloride (CsCl) density gradient ultra-centrifugation. The rAAV was then removed, desalted, filter-sterilized, titered using quantitative real-time PCR on a light cycler system (Roche) and resolved on SDS-PAGE gels. The titer of AAV-St8sia3 was 8.3×10^{13} vg/mL and the titer of AAV-hrGFP was 2.6×10^{13} vg/mL. rAAV vectors were diluted with 0.9% saline, aliquoted and stored at -80°C.

Receptor binding assay

Receptor binding assay was performed as previously described[7]. For the binding assay of

$A_{2A}R$, membrane proteins collected from mouse striatum were incubated in binding buffer (50 mM Tris-HCl, 10 mM $MgCl_2$, 1 mM EDTA, 1 U/mL adenosine deaminase and 0.25% Bovine serum albumin, pH 7.4) containing [^{3}H] CGS 21680 (0.05 μM, NET-1021, PerkinElmer) and the indicated concentration of cold CGS 21680 in a 250-μL reaction buffer, and incubated for 90 minutes at 25°C. For the binding assay of D_2R, striatal membranes were incubated in binding buffer containing Methoxy [^{3}H]-Sulpride (2 nM, NET-775, PerkinElmer) and the indicated concentration of cold Sulpride. Reactions were terminated by placing samples on ice and immediately filtering the reaction mixture through glass fiber filters (GF/C) in a vacuum filtration manifold system (Millipore). The membranes were washed with 5 mL ice-cold wash buffer (20 mM Tris and 0.25% BSA, pH 8.0) for 3 times and dried. The radioactivity on each GF/C membrane was determined in ULTIMA GOLD liquid scintillation cocktail (PerkinElmer) using the LS6500 Liquid Scintillation Counter (Beckman Coulter, USA).

Adenylyl cyclase (AC) assay

AC activity was assayed as previously described[8]. Briefly, striatum was homogenized using 2-mL glass grinder in lysis buffer (25 mM Tris, pH 8.0, 1 mM EGTA, 1 mM $MgCl_2$, 100 μM phenylmethylsulfonyl fluoride, 10 nM okadaic acid, 40 μM leupeptin, and 1x cOmplete™, EDTA-free Protease Inhibitor (Roche), 1x phosStop (Roche)). The homogenate was centrifuged at 500 × *g* for 10 minutes at 4°C to remove debris. AC activities were performed at 37°C by incubating striatal lysates (2 μg) for 10 minutes in reaction mixture (1 mM ATP, 100 mM NaCl, 50 mM HEPES, 0.5 mM isobutylmethylxanthine, 6 mM $MgCl_2$, 1 μM GTP, 0.2 mM EGTA). Reactions were terminated by 10% trichloroacetic acid. The cAMP formed was isolated by Dowex chromatography (Sigma-Aldrich) and assayed by a radioimmunoassay as described

previously[9].

In Situ Proximity Ligation Assay (PLA).

The *in situ* PLA was performed as reported previously[10-12]. The brain sections were incubated with anti-$A_{2A}R$ (1:750; Frontier Institute, Shinko-nishi, Ishikari, Hokkaido, Japan) and anti-D_2R (1:750; Frontier Institute) antibodies, followed by incubation with Duolink PLA probes (Sigma-Aldrich, St. Louis, MO, USA) and the Duolink detection reagent kit (Sigma-Aldrich). The PLA signal was imaged and quantified by using a Zeiss LSM780 confocal microscope system (Carl Zeiss, Jena, Germany) and the MetaMorph analysis (Molecular Devices).

Supplementary Results

Generation and characterization of *St8sia3*-KO mice.

The DNA sequence containing the putative start codon of the mouse *St8sia3* gene was selected as the targeting site for CRISPR/Cas9 (Clustered Regular Interspaced Short Palindromic Repeats/CRISPR-Associated Proteins 9)-mediated cleavage to generate a *St8sia3*-targeted allele (Supplementary Fig. S1A). As a result, 3 of 76 founders were confirmed to carry the expected indel mutations of *St8sia3* (Supplementary Fig. S1B). Heterozygous offspring were intercrossed to generate homozygous knockout pups (KO, designated *St8sia3*$^{-/-}$) and their wild-type littermate controls (WT, designated *St8sia3*$^{+/+}$). *St8sia3*-KO mice had no detectable *St8sia3* transcripts in the striatum (Supplementary Fig. S1C-D). The embryonic viability and fertility of *St8sia3*-KO mice were normal (Supplementary Table 3, 4). In addition, the absence of *St8sia3* did not up-regulate the expression of other *St8sia* enzymes in the striatum (Supplementary Fig. S2). A battery of behavioral phenotype tests was employed, and *St8sia3*-KO mice did not exhibit apparent abnormities in muscle, cerebellar, sensory, neuropsychiatric and autonomic functions compared with their WT controls (Supplementary Fig. S3A-H, Supplementary File 1). We performed the rotarod test to examine motor coordination (Supplementary Fig. S3I). *St8sia3*-KO mice exhibited poorer performance than WT mice in all three trials, suggesting that the absence of ST8SIA3 impaired motor coordination.

Supplementary Table 1. The primary antibodies used in this study.

Name	Antibody
	For immunofluorescent staining
ST8SIA3	rabbit polyclonal antibody_#ab222305_abcam_1:500
NeuN	mouse monoclonal antibody_#MAB377_Millipore_1:500
S100 beta	mouse monoclonal antibody_#AMAB91038_Sigma-Aldrich_1:500
Iba1	mouse monoclonal antibody_#GTX632426_GeneTex_1:500
$A_{2A}R$	goat polyclonal antibody_#Af700_Frontier Institute_1:500
D_1R	goat polyclonal antibody_#Af1000_Frontier Institute_1:500
D_2R	guinea pig polyclonal antibody_#Af500_Frontier Institute_1:500
	For immunoblotting
ST8SIA3	rabbit polyclonal antibody_#ab222305_abcam_1:3000
AC5	rabbit polyclonal antibody_1:5000 (Homemade)
$A_{2A}R$	mouse monoclonal antibody[7F6-G5-A2]_#sc-32261_Santa Cruz_1:1000
D_1R	goat polyclonal antibody_#Af1000_Frontier Institute_1:3000
D_2R	guinea pig polyclonal antibody_#Af500_Frontier Institute_1:3000
NR1	rabbit monoclonal antibody (D65B7)_#5704_Cell Signaling_1:1000
NR2A	rabbit polyclonal antibody_#AB1555P_Millipore_1:500
NR2B	rabbit monoclonal antibody (D15B3)_#4212S_Cell Signaling_1:1000
GluR1	rabbit polyclonal antibody_#AB1504_Millipore_1:1000
GluR2	mouse monoclonal antibody [6C4]_#MAB397_Millipore_1:2000
S2-566	mouse monoclonal antibody_1:1000 (provided from Dr. Chihiro SATO)
12E3	mouse monoclonal antibody_1:1000 (provided from Dr. Chihiro SATO)

A2B5	mouse monoclonal antibody [105]_#GTX14538_GeneTex_1:1000
PSA	mouse monoclonal antibody [735]_#Ab00240-2.0_absolute_1:500
TUBULIN	mouse monoclonal antibody [GT114]_#GTX628802_GeneTex_1:10000
ACTIN	rabbit polyclonal antibody_#A2066_Sigma-Aldrich_1:10000
Na^+/K^+ ATPase	mouse monoclonal antibody_#ab7671_Abcam_1:3000
FLOT1	rabbit polyclonal antibody_#GTX104769_ GeneTex_1:3000

Supplementary Table 2. **The glycan list for microarray fabrication and detection.**

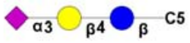<br>**2**	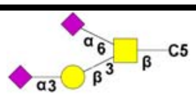<br>**15**	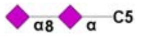<br>**20**
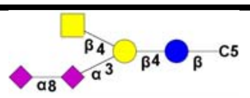<br>**31**	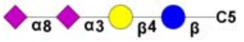<br>**32**	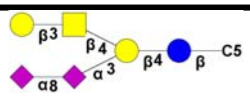<br>**34**
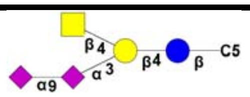<br>**54**	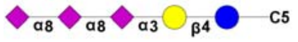<br>**204**	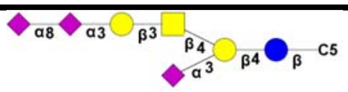<br>**205**
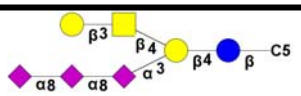<br>**206**	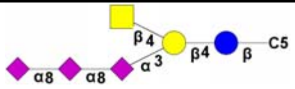<br>**207**	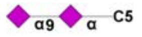<br>**PS1**
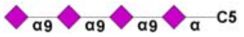<br>**PS2**	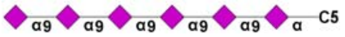<br>**PS3**	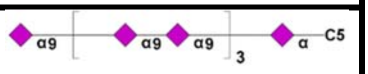<br>**PS4**
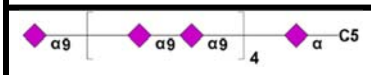<br>**PS5**	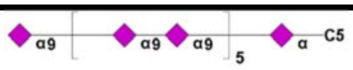<br>**PS6**	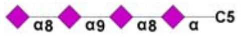<br>**PS7**
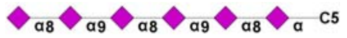<br>**PS8**	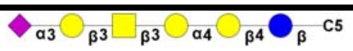<br>**12**	

Supplementary Table 3. Summary of offspring produced from an intercross between *St8sia3*$^{+/-}$ males and *St8sia3*$^{+/-}$ females designed to examine embryonic viability.

Age	Total offspring/litter (mean litter size)	No. of offspring with the *St8sia3* genotype		
		+/+	+/-	-/-
P80	128/20 (6.4 pups/litter)	30 (23%)	65 (51%)	33 (26%)
Expected Mendelian Ratio		*32 (25%)*	*64 (50%)*	*32(25%)*

P: postnatal day

Supplementary Table 4. Summary of offspring produced from an intercross between *St8sia3*$^{-/-}$ males and *St8sia3*$^{-/-}$ females designed to examine adult fertility.

Age	Total offspring/litter	Mean litter size (-/-)
P80	68/10	6.8
Expected Litter Size		*6*[13]

P: postnatal day

Supplementary Table 5. The primers of qRT-PCR used in this study.

Name	Primer Sequence
St8sia1-F	5' TGGGATTCTGAAGATGAGTGG 3'
St8sia1-R	5' CTTCTTTCTGGACCACAGCAG 3'
St8sia2-F	5' GACACAACCAGACGCTCTCTC 3'
St8sia2-R	5' TGGGGAGGAGTTCATAGAGGT 3'
St8sia3-3FP	5' TCTTCACCACTCCCAAGTACG 3'
St8sia3-4RP	5' GAGAATTCGTAATGGGCACAA 3'
St8sia4-F	5' CGGAGATGGTGAACTGTGTTT 3'
St8sia4-R	5' TCCGCATCTAAGAAACGAAGA 3'
St8sia5-F	5' ATGCCCTACTACCGCTCTCA 3'
St8sia5-R	5' GTTGCACCGGAAGACAAAGT 3'
St8sia6-F	5' TGGTTTCCCAGAACAACACTC 3'
St8sia6-R	5' GGGGAGGTTACACCTGAAGAC 3'

Supplementary File 1. Analysis of the results from the modified SHIRPA protocol for WT and *St8sia3*-KO mice.

A battery of behavioral tests was employed to assess the behavioral phenotypes of *St8sia3*-KO mice. The modified SHIRPA protocol is composed of fifty-eight observations designed to assess muscle, cerebellar, sensory, neuropsychiatric and autonomic functions [3, 4]. When compared with their littermate WT controls, *St8sia3*-KO mice appeared normal without apparent abnormities.

Supplementary References

1. Yang H, Wang H, Jaenisch R. Generating genetically modified mice using CRISPR/Cas-mediated genome engineering. *Nature protocols* 2014; **9**(8)**:** 1956-1968.

2. Bermejo MK, Milenkovic M, Salahpour A, Ramsey AJ. Preparation of synaptic plasma membrane and postsynaptic density proteins using a discontinuous sucrose gradient. *J Vis Exp* 2014;(91)**:** e51896.

3. Rogers DC, Fisher EM, Brown SD, Peters J, Hunter AJ, Martin JE. Behavioral and functional analysis of mouse phenotype: SHIRPA, a proposed protocol for comprehensive phenotype assessment. *Mammalian genome : official journal of the International Mammalian Genome Society* 1997; **8**(10)**:** 711-713.

4. Rogers DC, Jones DN, Nelson PR, Jones CM, Quilter CA, Robinson TL*, et al.* Use of SHIRPA and discriminant analysis to characterise marked differences in the behavioural phenotype of six inbred mouse strains. *Behavioural brain research* 1999; **105**(2)**:** 207-217.

5. Hsiao CT, Wang PW, Chang HC, Chen YY, Wang SH, Chern Y*, et al.* Advancing a High Throughput Glycotope-centric Glycomics Workflow Based on nanoLC-MS(2)-product Dependent-MS(3) Analysis of Permethylated Glycans. *Molecular & cellular proteomics : MCP* 2017; **16**(12)**:** 2268-2280.

6. Wang CC, Huang YL, Ren CT, Lin CW, Hung JT, Yu JC*, et al.* Glycan microarray of Globo H and related structures for quantitative analysis of breast cancer. *Proceedings of the National Academy of Sciences of the United States of America* 2008; **105**(33)**:** 11661-11666.

7. Huang NK, Lin JH, Lin JT, Lin CI, Liu EM, Lin CJ*, et al.* A new drug design targeting the adenosinergic system for Huntington's disease. *PloS one* 2011; **6**(6)**:** e20934.

8. Chien CL, Wu YS, Lai HL, Chen YH, Jiang ST, Shih CM*, et al.* Impaired water reabsorption in mice deficient in the type VI adenylyl cyclase (AC6). *FEBS Lett* 2010; **584**(13)**:** 2883-2890.

9. Chern Y, Lai HL, Fong JC, Liang Y. Multiple mechanisms for desensitization of A2a adenosine receptor-mediated cAMP elevation in rat pheochromocytoma PC12 cells. *Molecular pharmacology* 1993; **44**(5)**:** 950-958.

10. Borroto-Escuela DO, Wydra K, Li X, Rodriguez D, Carlsson J, Jastrzębska J*, et al.* Disruption of A2AR-D2R Heteroreceptor Complexes After A2AR Transmembrane 5 Peptide Administration Enhances Cocaine Self-Administration in Rats. *Molecular Neurobiology* 2018; **55**(8)**:** 7038-7048.

11. Borroto-Escuela DO, Narvaez M, Wydra K, Pintsuk J, Pinton L, Jimenez-Beristain A*, et*

al. Cocaine self-administration specifically increases A2AR-D2R and D2R-sigma1R heteroreceptor complexes in the rat nucleus accumbens shell. Relevance for cocaine use disorder. *Pharmacol Biochem Behav* 2017; **155:** 24-31.

12. Borroto-Escuela DO, Hagman B, Woolfenden M, Pinton L, Jiménez-Beristain A, Oflijan J, *et al*. In Situ Proximity Ligation Assay to Study and Understand the Distribution and Balance of GPCR Homo- and Heteroreceptor Complexes in the Brain. In: Luján R, Ciruela F (eds). *Receptor and Ion Channel Detection in the Brain: Methods and Protocols*. Springer New York: New York, NY, 2016, pp 109-124.

13. Foldi CJ, Eyles DW, McGrath JJ, Burne TH. The effects of breeding protocol in C57BL/6J mice on adult offspring behaviour. *PloS one* 2011; **6**(3)**:** e18152.

Supplementary Figure Legends

Supplementary Fig. S1. *St8sia3* targeting strategy and CRISPR/Cas9-mediated genome engineering.

(**a**) Schematic representation of the *St8sia3* gene-targeting strategy. Exons with untranslated regions (white boxes) and translated sequences (black boxes) are indicated. Most of exon 3, including the translation start codon (ATG), and all of exon 4 were deleted. The binding sites of the primers used for genotyping and RT-PCR are indicated as arrows. (**b**) Sequences of the *St8sia3*-targeted alleles in the three founder mice. PCR amplicons carrying targeted sequences from genomic DNA obtained from tail biopsies were sub-cloned and sequenced. Mouse #16 carried one allele with a 1966 base pair (bp) deletion and an 18 bp insertion. Mouse #49 carried one allele with a 1966 bp deletion and a 4 bp insertion. Mouse #59 carried one allele with a 1958 bp deletion and no insertion. Three male founders were confirmed to carry *St8sia3*-targeted alleles and all *St8sia3* mutations disrupted the translation start site (ATG deletion). (**c**) PCR genotyping. Genomic DNA was obtained from the WT (+/+) and *St8sia3*-KO (-/-) mice from three lines (#16, #49 and #59). WT alleles generated 515-bp products and targeted alleles yielded 295-, 301- and 312-bp products. (**d**) Detection of *St8sia3* transcripts by RT-PCR. The mRNA was extracted from the striatum of WT and *St8sia3*-KO mice. WT mice exhibited a 123-bp product corresponding to *St8sia3* exons 3 and 4. In contrast, this product was not detected in *St8sia3*-KO mice. *Gapdh* was as a positive control for cDNA production. (**e**) The sections were costained for ST8SIA3 (green) and cell-specific markers (red) to identify astrocytes (S100 beta) and glial cells (Iba1). The nuclei are marked by Hoechst 33342 (blue). ST8SIA3 was not detected in S100 beta-positive astrocytes or Iba1-positive glial cells. The data shown are representative of three mice of each genotype. Scale bar, 10 µm.

Supplementary Fig. S2. Relative mRNA expression levels of the *St8sia* family in the striatum.

Relative mRNA expression levels of the *St8sia* family, including *St8sia3* (**a**), *St8sia1* (**b**), *St8sia2* (**c**), *St8sia4* (**d**), *St8sia5* (**e**) and *St8sia6* (**f**) transcripts, were measured by quantitative real time reverse transcriptase PCR. (**a**) Quantitative PCR did not detect the *St8sia3* transcripts in *St8sia3*-KO (-/-) striatum. (**d**) *St8sia4* expression was significantly decreased in *St8sia3*-KO mice compared with WT (+/+) mice. The levels of the other four *St8sia* transcripts were comparable between the two genotypes. n = 4 for each genotype with triplicates. ***P<0.001 and *P<0.05, compared with WT control (two-tailed unpaired Student's t-test). All values are presented as the means ± S.E.M. The primers were listed in Supplementary Table 5.

Supplementary Fig. S3. The absence of ST8SIA3 impaired motor coordination determined by rotarod test.

Behavioral observations, including developmental and neurophysiological screens, were compared between WT (+/+) and *St8sia3*-KO (-/-) mice. (**a**) Body weights of 12-week-old mice. No gross developmental abnormalities were observed between genotypes. (**b, c**) Open field test. No differences between genotypes were observed in the locomotor activity over 30 minutes, as evidenced by the similar horizontal and vertical counts. (**d**) Grip strength. No significant deficit in grip strength was observed between genotypes. (**e-h**) Thermal nociception and pain sensitivity as an assessment of the sensory system. *St8sia3*-KO mice showed no significant changes in the hot plate (**e**, **f**), tail flick (**g**) and electronic von Frey tests (**h**) compared with WT mice. n = 10 for each genotype. Statistical analyses were performed using the two-tailed unpaired Student's t test.

(**i**) The rotarod test detected a decreased latency for *St8sia3*-KO mice to stay on the rotarod compared with that of WT mice throughout three trials with 15 minute-intervals. n = 10 for each genotype. **P<0.01 and ***P<0.001, compared with WT control (two-way ANOVA followed by Bonferroni's multiple comparison tests). All values are presented as the means ± S.E.M.

Supplementary Fig. S4. *St8sia3* depletion impaired the addition of terminal sialylated units to striatal *O*-glycans.

Permethylated reduced *O*-glycans derived from WT and *St8sia3*-KO mice were subjected to nanoLC-MS/MS analysis. (**a**) Overlaid extracted ion chromatograms (XIC) of either the singly or doubly charged MS ions representing each of the isomeric *O*-glycans detected, as annotated by the cartoon symbols. (**b**) Relative quantification of each of the detected *O*-glycans based on the area under the XIC. The data included peaks assigned as NeuAc-Hexitol (*m/z* 628.3) and $NeuAc_2itol$ (*m/z* 785.3), which are likely fragments derived from larger glycans that were produced during sample preparation. A smaller amount of the highly sialylated version of each product was also detected, quantified and expressed as relative ratios in WT versus *St8sia3*-KO mice (inset in **b**). (**c**) The presence of the assigned *O*-glycans was supported by their respective MS/MS data, which also allowed us to discriminate between the two chromatographically resolved, trisialylated core 1 *O*-glycans, as shown for the earlier (left) and later (right) eluting peaks. n = 3 for each genotype with duplicates. *P<0.05, compared with WT control (two-tailed unpaired Student's *t*-test). All values are presented as the means ± S.E.M.

Supplementary Fig. S5. ST8SIA3 deficiency affected diSIA and triSIA formation in different brain regions.

Disialyl (**a**) and trisialyl (**b**) units were detected in the brain lysates. Immunoblotting revealed reductions in diSia-Gal and triSia terminal glycotopes in the striatum, hippocampus, cortex and cerebellum of *St8sia3*-KO (-/-) mice compared with those in WT (+/+) control. $n = 3$ for each genotype. TUBULIN was used as an internal loading control.

Supplementary Fig. S6. Genetic ablation of *St8sia3* did not affect the sialylation of ionotropic glutamate receptors.

Ionotropic glutamate receptors (iGluRs) are ion channel-associated proteins that are activated by glutamate. (**a**) Similar sizes of the NR1 protein, an NMDA receptor subunit, were observed in the striatal lysates from WT (+/+) and *St8sia3*-KO (-/-) mice. (**b, c**) Equivalent sizes of the NR2A and NR2B NMDA receptor subunits were observed in the striatum of the *St8sia3*-KO and WT mice. (**d, e**) Striatal lysates from *St8sia3*-KO and WT mice exhibited similarly sized bands for the GluR1 and GluR2 subunits of the AMPA receptor. $n = 3$ for each genotype. ACTIN was used as an internal loading control.

Supplementary Fig. S7. Intrastriatal delivery of AAV serotype 8 in the mouse brain.

AAV serotype 8 (AAV8) vectors carrying the enhanced green fluorescent protein (eGFP) as a reporter gene driven by the CAG promoter were injected directly into the mouse striatum. Representative fluorescence images from injected mice showed the high efficiency of transgene transduction in the striatum. Scale bar, 100 μm.

Supplementary Fig. S8. Double immunofluorescence staining of ST8SIA3 and $A_{2A}R$, D_1R or D_2R in the striatum of wild-type mice.

Representative images of ST8SIA3 expression accompanied by $A_{2A}R$, D_1R and D_2R immunoreactivities in the striatum of WT mice. The sections were costained for ST8SIA3 (red) and three GPCRs (green). $n = 3$ for each genotype. Scale bars, 100 μm for 10x images; 10 μm for 63x images; 5 μm for enlarged images.

Supplementary Fig. S9. The ablation of ST8SIA3-mediated sialylation did not affect the trafficking of ST8SIA3 substrates.

Extracted striatal proteins from WT (+/+) and *St8sia3*-KO (-/-) mice were separated into three fractions by centrifugation: total lysates, membrane fractions and cytosolic fractions. The ablation of ST8SIA3-mediated sialylation did not affect the trafficking of AC5, $A_{2A}R$, D_2R, and D_1R in the striatum. $n = 3$ for each genotype.

Supplementary Fig. S10. The deficiency of ST8SIA3-mediated sialylation did not affect the synaptic distribution of AC5, $A_{2A}R$, D_2R and D_1R in the striatum.

Striatal synaptosomes, isolated synaptic terminals from neurons from WT (+/+) and *St8sia3*-KO (-/-) mice were prepared using a discontinuous sucrose gradient[2]. (**a**) Extracted proteins were separated into five fractions by centrifugation: total lysates (Total), cytosolic fractions (Cytosol), crude synaptosomal fractions (Crude SYN), crude synaptic vesicles (Crude SV) and synaptosome (SYN). PSD95 and syntaxin-1 were used as internal controls for synaptic fractions. (**b, c**) The ablation of ST8SIA3-mediated sialylation did not affect the synaptic distribution of AC5, $A_{2A}R$, D_2R, or D_1R in the striatum. Accordingly, these proteins were all enriched in the synaptic plasma membrane. $n = 6$ for each genotype with three independent experiments (two-tailed unpaired Student's *t*-test). All values are presented as the means ±

S.E.M.

Supplementary Fig. S11. *St8sia3* disruption did not change the lipid raft distribution of ionotropic glutamate receptors.

Lipid raft membrane regions from the striatum of WT (+/+) and *St8sia3*-KO (-/-) mice were isolated using a nondetergent method and analyzed for protein distribution by immunoblotting. NR1 (**a**), NR2A (**b**), NR2B (**c**), GluR1 (**d**) and GluR2 (**e**) were all dispensed in fraction 3 (raft) along with FLOT1, a well-characterized lipid raft marker. The ST8SIA3 deficiency did not alter the lipid raft distribution of NR1, NR2A, NR2B, GluR1 and GluR2. $n = 3$ for each genotype.

Supplementary Fig. S12. Increased AC5, A_{2A}R, D_1R and D_2R in lipid rafts purified from the striatum of *St8sia3*-KO mice.

Lipid raft and nonraft membrane regions were isolated using a nondetergent method and analyzed for the distributions of ST8SIA3 substrates and FLOT1 (a raft marker) by immunoblotting. Fourteen fractions were collected from the top to bottom. Increasing distributions of AC5, A_{2A}R (**a**), D_1R (**b**) and D_2R (**c**) were found in lipid rafts. No significant changes were observed in the amounts of NR2A, NR1 (**d**), GluR1 (**e**), NR2B and GluR2 (**f**) in all regions. $n = 3$ for each genotype.

Supplementary Fig. S13. *St8sia3* ablation enhanced the formation of A_{2A}R-D_2R heteromers in the striatum.

(**a**) Detection of A_{2A}R-D_2R heteroreceptor complexes in the dorsal striatum using the *in situ* PLA. Representative images indicated increased densities of A_{2A}R-D_2R-positive blobs in the

striatum of *St8sia3*-KO (-/-) mice compared with WT (+/+) mice. Nuclei were stained with DAPI in blue. The left column shows A_{2A}R-D_2R-positive PLA blobs in red, the middle column shows merged images, and the right column shows enlarged regions. Scale bars, 20 μm in merged images, 5 μm in enlarged images. (**b**) *St8sia3*-KO mice exhibited a significant increase in the average number of PLA blobs per field. *n* = 9 for each genotype with three independent fields. ****P*<0.001, compared with WT control (two-tailed unpaired Student's *t*-test). All values are presented as the means ± S.E.M.

Supplementary Fig. S14. *St8sia3* depletion did not alter the binding properties of A_{2A}R and D_2R or adenylyl cyclase (AC) activity.

Plasma membrane fractions were collected from the striatum of *St8sia3*-KO and wild-type (WT) mice. (**a**) Binding of CGS 21680, an A_{2A}R-selective agonist, to striatal membranes was carried out as described. The B_{Max} of WT striatum (100%) was 384.9 ± 66.8 nmol/mg. The estimated Ki values of the WT and *St8sia3*-KO striatum are 0.06 and 0.04 μM, respectively. No significant difference was observed between these two groups. *n* = 30 for each genotype from four independent experiments. (**b**) Binding of Sulpiride, a selective dopamine D_2-like receptor antagonist, to striatal membranes was performed as described. The B_{Max} of WT striatum (100 %) was 4450.0 ± 151.5 fmol/mg. The estimated Ki values for the WT and *St8sia3*-KO striatum are 0.04 and 0.02 μM, respectively. No significant difference was observed between two groups. *n* = 30 for each genotype from four independent experiments. (**c**) Striatal AC activities evoked by forskolin, a direct activator of AC, were measured as described. The AC activity of WT striatum evoked by 100 μM forskolin (100%) was 1499.1 ± 206.4 pmol/mg/min. The estimated EC_{50} values for the striatal membranes purified from WT and *St8sia3*-KO mice are 2.21 and 2.69 μM,

respectively. No significant difference was observed between two groups. n = 10 mice for each genotype. Two-way ANOVA (genotype vs. dose) was performed using the Holm-Sidak method. All values are presented as the means ± S.E.M.

Supplementary Fig. S15. The *St8sia3*-deficient mice exhibited alterations in the locomotor response to the receptor-selective modulators.

(**a-c**) Mice (male, 8-12 weeks old) were injected with saline or various doses of receptor-selective modulators, placed individually into chambers, and locomotor activity was determined by counting the photobeam breaks for 1 hour. (**a**) Effects of SCH 58261, an $A_{2A}R$-selective antagonist. *St8sia3*-KO (-/-) mice showed less of a response to SCH 58261, resulting in lower activity at higher doses (1-10 mg/kg), than WT (+/+) mice. (**b**) Responses to L-741626, a D_2R-selective antagonist. *St8sia3*-KO mice showed greater responses to L-741626, leading to rapidly diminished motor performance at lower doses (<5 mg/kg) than WT mice. (**c**) Reactions to SKF 81297, a D_1R-selective agonist. After the SKF 81297 injection, the *St8sia3*-KO mice did not exhibit a noticeable locomotor response to drugs at the doses (1-10 mg/kg) that affected WT mice. Horizontal axes indicate the drug dose in mg/kg. Vertical bars indicate locomotor activity (beam breaks). n = 8 mice for each genotype were administered each drug at 10-12 weeks of age. (**d-f**) WT and *St8sia3*-KO male mice were treated with intrastriatal injection of AAV virus expressing mouse ST8SIA3 (AAV-St8sia3) or hrGFP control (AAV-hrGFP) at 5 weeks of age. (**d**) AAV-St8sia3 rescued the slight response of *St8sia3*-KO mice to 1 mg/kg SCH 58261 to the original level. (**e**) Treatment with AAV-St8sia3 resulted in a significant recovery of the diminished motor performance induced by 0.5 mg/kg L-741626. (**f**) The AAV-St8sia3 infection enabled the *St8sia3*-KO mice to respond to 5 mg/kg SKF 81297. n = 6 mice per

genotype. Two-way ANOVA (genotype vs. virus) revealed a major genotype effect and virus effect for viruses and both groups. *Post hoc* Bonferroni's test showed significant differences between the groups at the indicated doses. *$P < 0.05$, **$P < 0.01$ and ***$P < 0.001$. All values are presented as the means ± S.E.M.
