# Supplementary material for: Functional roles of ST8SIA3-mediated sialylation of striatal dopamine D2 and adenosine A2A receptors
Source: Transl Psychiatry. 2019 Aug 27;9:209. doi: 10.1038/s41398-019-0529-z (PMC6712005; doi:10.1038/s41398-019-0529-z)
Supplement: Supplementary file 2 — Supplementary File [file 41398_2019_529_MOESM2_ESM.pdf]

File S1. Analysis of the results from the modified SHIRPA protocol for WT and *St8sia3* -KO mice.

Modified-SHIRPA of St8sia3 WT/KO mice

List of tests in modified-SHIRPA (Individual score for each mouse)

The scores in black indicate criteria by C5BL/6J mice

Items	Score	Description	*St8sia3* WT Mice										*St8sia3* KO Mice									
			#1	#2	#3	#4	#5	#6	#7	#8	#9	#10	#1	#2	#3	#4	#5	#6	#7	#8	#9	#10
1.Coat color	0=<br>1=<br>2=<br>3=<br>4=	Normal<br>Dilution<br>Whitespot<br>Color change<br>Other	0	0	0	0	0	0	0	0	0	0	0	0	0	0	0	0	0	0	0	0
2.Hair length	0=<br>1=<br>2=<br>3=	Normal<br>alopecia<br>short<br>long	0	0	0	0	0	0	0	0	0	0	0	0	0	0	0	0	0	0	0	0
3.Hair morphology	0=<br>1=<br>2=	Normal<br>Curl<br>Other	0	0	0	0	0	0	0	0	0	0	0	0	0	0	0	0	0	0	0	0
4.Respiration rate	0=<br>1=<br>2=<br>3=	Gasping, irregular<br>Slow, shallow<br>Normal<br>Hyperventilation	2	2	2	2	2	2	2	2	2	2	2	2	2	2	2	2	2	2	2	2
5.Tremor	0=<br>1=<br>2=	None<br>Mild<br>Markerd	0	0	0	0	0	0	0	0	0	0	0	0	0	0	0	0	0	0	0	0
6.Body position	0=<br>1=<br>2=<br>3=<br>4=<br>5=	Completely flat<br>Lying on side<br>Lying prone<br>Sitting or standing<br>Rearing on hind legs<br>Repeated vertical leaping	3	3	3	3	3	3	3	3	3	3	3	3	3	3	3	3	3	3	3	3
7. Spontaneous activity	0=<br>1=<br>2=<br>3=<br>4=	None, resting<br>Casual scratch, groom, slow movement<br>Vigorous scratch, groom, moderate movement<br>Vigorous, rapid/dart movement<br>Extremely vigorous, rapid/dart movement	2	2	2	2	2	2	2	2	2	2	2	2	2	2	2	2	1	1	1	2
8.Defecation			3	3	3	4	2	3	2	2	3	2	2	1	2	0	2	2	5	2	5	1
9.Urination	0=<br>1=	None<br>Done	1	0	0	0	0	0	0	0	0	0	0	0	0	0	0	0	0	0	0	0
10.Elapsed time before the mouse starts to move (secs)			2	2	4	3	5	3	4	4	3	4	3	3	3	2	6	3	3	6	5	4
11.Locomotor acitivity			18	21	22	21	12	19	15	18	22	16	19	25	20	20	16	11	18	4	16	15
12.transfer arousal	0=<br>1=<br>2=<br>3=<br>4=<br>5=	Coma<br>Prolonged freeze, then slight movement<br>Extended freeze, then moderate movement<br>Brief freeze (few seconds), then active movement<br>Momentary freeze, then swift movement<br>No freeze, immediate movement	3	3	3	3	3	3	3	3	3	3	3	3	3	3	3	3	3	3	3	3
13.Piloerection	0=<br>1=	None<br>Coat stood on end	0	0	0	0	0	0	0	0	0	0	0	0	0	0	0	0	0	0	0	0
14.Palpebral closure	0=<br>1=<br>2=	Eyes wide open<br>Eyes 1/2 closed<br>Eyes closed	0	0	0	0	0	0	0	0	0	0	0	0	0	0	0	0	0	0	0	0
15.Startle response	0=<br>1=<br>2=<br>3=	None<br>Preyer reflex (backwards flick of pinnae)<br>Jump less than 1cm<br>Jump more than 1cm	1	1	1	1	1	1	1	1	1	1	1	1	1	1	1	1	1	1	1	1
16.Gait	0=<br>1=<br>2=<br>3=	Normal<br>Fluid but abnormal<br>Limited movement only<br>Incapacity	0	0	0	0	0	0	0	0	0	0	0	0	0	0	0	0	0	0	0	0
17.Pelvic elevation	0=<br>1=<br>2=<br>3=	Markedly flattened<br>Barely touches<br>Normal (3mm elevation)<br>Elevated (more than 3mm elevation)	2	2	2	2	2	2	2	2	2	2	2	2	1	2	2	2	2	2	2	2
18.Tail elevation	0=<br>1=<br>2=	Dragging<br>Horizontally extended<br>Elevated/Straub Tail	1	1	1	1	1	1	1	1	1	1	1	1	1	1	1	1	1	1	1	1
19.Touch escape	0=<br>1=<br>2=<br>3=	No response<br>Mild (escape response to firm stroke)<br>Moderate (rapid response to light stroke)<br>Vigorous (escape response to approach)	2	2	2	2	2	2	2	2	2	2	2	2	2	2	2	2	2	2	2	2
20.Positional passivity	0=<br>1=<br>2=<br>3=<br>4=	Struggles when held by tail<br>Struggles when held by neck (finger grip, not scruf<br>Struggles when laid supine (on back)<br>Struggles when held by hind legs<br>No straggle	0	0	0	0	0	0	0	0	0	0	0	0	0	0	0	0	0	0	0	0
21.Trunk curl	0=<br>1=	Absent<br>Present	0	0	0	0	0	0	0	0	0	0	0	0	0	0	0	0	0	0	0	0
22.Limb grasping	0=<br>1=	Absent<br>Present	0	0	0	0	0	0	0	0	0	0	0	0	0	0	0	0	0	0	0	0
23.Visual Placing	0=<br>1=<br>2=<br>3=<br>4=	None<br>Upon nose contact<br>Upon vibrasse contact<br>Before vibrasse contact (18mm)<br>Early vigorous extension (25mm)	2	2	2	2	2	2	2	2	2	2	2	2	2	2	2	2	2	2	2	2
24.Grip strength	0=<br>1=<br>2=<br>3=<br>4=	None<br>Slight grip, semi-effetive<br>Moderate grip, effective<br>Active grip, effective<br>Unusually effective	1	1	1	1	1	1	1	1	1	1	1	1	1	1	1	1	1	1	1	1
25.Body Tone	0=<br>1=<br>2=	Flaccid, no return of cavity to normal<br>Slight resistance<br>Extreme resistance, board like	1	1	1	1	1	1	1	1	1	1	1	1	1	1	1	1	1	1	1	1
26.Head morphology	0=<br>1=	Normal<br>Abnormal	0	0	0	0	0	0	0	0	0	0	0	0	0	0	0	0	0	0	0	0
27.Pinna reflex	0=<br>1=<br>2=	None<br>Active retraction, moderately brisk flick<br>Hyperactive, repetitive flick	1	1	1	1	1	1	1	1	1	1	1	1	1	1	1	1	1	1	1	1
28.Pinna morphology(R)	0=<br>1=	Normal<br>Abnormal	0	0	0	0	0	0	0	0	0	0	0	0	0	0	0	0	0	0	0	0
29.Pinna morphology(L)	0=<br>1=	Normal<br>Abnormal	0	0	0	0	0	0	0	0	0	0	0	0	0	0	0	0	0	0	0	0
30.Corneal reflex	0=<br>1=<br>2=	None<br>Active single eye blink<br>Multiple eye blink	1	1	1	1	1	1	1	1	1	1	1	1	1	1	1	1	1	1	1	1
31.Toe pinch	0=<br>1=<br>2=<br>3=<br>4=	None<br>Slight withdrawal<br>Moderate withdrawal, not brisk<br>Brisk, rapid withdrawal<br>Very brisk repeated extension and flexion	3	3	3	3	3	3	3	2	3	3	3	1	3	3	3	3	3	3	3	3
32.Body length (mm)			94	96	96	95	93	94	95	91	96	95	93	95	92	92	92	95	92	95	90	92
33.Tail length (mm)			86	86	86	86	83	88	89	87	88	87	88	90	87	89	87	90	90	91	89	89
34.Tail morphology	0=<br>1=<br>2=<br>3=	Normal<br>Kink<br>Curl<br>Other	0	0	0	0	0	0	0	0	0	0	0	0	0	0	0	0	0	0	0	0
35.Lacrimation	0=<br>1=	None<br>Present	0	0	0	0	0	0	0	0	0	0	0	0	0	0	0	0	0	0	0	0
36.Whisker morphology	0=<br>1=	Normal<br>Abnormal	0	0	0	0	0	0	0	0	0	0	0	0	0	0	0	0	0	0	0	0
37.Teeth morphology	0=<br>1=	Normal<br>Abnormal	0	0	0	0	0	0	0	0	0	0	0	0	0	0	0	0	0	0	0	0
38.Provoked biting	0=<br>1=	Absent<br>Present	1	1	1	1	1	1	1	1	1	1	1	1	1	1	1	1	1	1	1	1
39.Salivation	0=<br>1=<br>2=	None<br>Slight margin of sub-maxillary area<br>Wet zone entire sub-maxillary area	1	1	1	1	1	1	1	1	1	1	1	1	1	1	1	1	1	1	1	1
40.Heart Rate	0=<br>1=<br>2=	Slow, bradycardia<br>Normal<br>Fast, tachycardia	1	1	1	1	1	1	1	1	1	1	1	1	1	1	1	1	1	1	1	1
[illegible]	0=	Flaccid, no return of cavity to normal																				

41. Abdominal tone	1=	Slight resistance	1	1	1	1	1	1	1	1	1	1	1	1	1	1	1	1	1	1	1	1
	2=	Extreme resistance, board like																				
42.Skin color	0=	Blanched	1	1	1	1	1	1	1	1	1	1	1	1	1	1	1	1	1	1	1	1
	1=	Pink																				
	2=	Bright, deep red flush																				
	3=	Mild pigmentation																				
	4=	Dark footpad, pigmentation																				
43.Limb morphology(FR)	0=	Normal	0	0	0	0	0	0	0	0	0	0	0	0	0	0	0	0	0	0	0	0
44.Limb morphology(FL)	1=	Polydactyly	0	0	0	0	0	0	0	0	0	0	0	0	0	0	0	0	0	0	0	0
45.Limb morphology(HR)	2=	Ectodactyly	0	0	0	0	0	0	0	0	0	0	0	0	0	0	0	0	0	0	0	0
46.Limb morphology(HL)	3=	Abnormality in upper limb	0	0	0	0	0	0	0	0	0	0	0	0	0	0	0	0	0	0	0	0
	4=	Other abnormality																				
47.Limb tone	0=	No resistance	1	1	1	1	1	1	1	1	1	1	1	1	1	1	1	1	1	1	1	1
	1=	Slight resistance																				
	2=	Moderate resistance																				
	3=	Marked resistance																				
	4=	extreme resistance																				
48. Wire maneuver	0=	Active grip with hindlegs (5secs)	0	0	0	0	1	0	0	0	0	0	0	1	1	0	0	1	2	1	2	0
	1=	Difficulty to grip with hindlegs																				
	2=	Unable to grip with hindlegs																				
	3=	Unable to lift hindlegs, falls within seconds																				
	4=	Falls immediately																				
49.Righting Reflex	0=	No, impairment	0	0	0	0	0	0	0	0	0	0	0	0	0	0	0	0	0	0	0	0
	1=	Lands on side																				
	2=	Lands on back																				
	3=	Fails to right when placed on back																				
50.Contact righting reflex	0=	Absent	1	1	1	1	1	1	1	1	1	1	1	1	1	1	1	1	1	1	1	1
	1=	Present																				
51.Negative geotaxis	0=	Turns and climbs the grid	0	0	0	0	0	0	0	0	0	0	0	0	0	0	0	0	0	0	0	0
	1=	Turns but then freezes																				
	2=	Moves, but fails to turn																				
	3=	Does not move within 30 secs																				
	4=	Falls off																				
52.Fear	0=	None	0	0	0	0	0	0	0	0	0	0	0	0	0	0	0	0	0	0	0	0
	1=	Freezes during transfer arousal																				
53.Irritability	0=	None	0	0	0	0	0	0	0	0	0	0	0	0	0	0	0	0	0	0	0	0
	1=	Struggle during spine restraint																				
54.Aggression	0=	None	0	0	0	0	0	0	0	0	0	0	0	0	0	0	0	0	0	0	0	0
	1=	Provoked biting or attack																				
55.Vocalization	0=	None	1	1	1	1	1	1	1	1	1	1	1	1	1	1	1	1	1	1	1	1
	1=	Provoked during handling																				
56.Bizarre Behaviour	0) No Phenotype		0	0	0	0	0	0	0	0	0	0	0	0	0	0	0	0	0	0	0	0
	a) HF	Head flicking or head shaking																				
	b) HS	Head searching - repetitive																				
	c) H	Hallucinating																				
	d) B	Compulsive biting - usually of grid floor																				
	e) L	Compulsive licking - usually of glass jar																				
	f) SB	Self destructive biting																				
	g) P	Prancing forelimbs																				
	h) UW	Upright walking - hind limbs only																				
	i) AW	Aimless wandering																				
	j) C	Circling																				
	k) W	Waltzing - rapid turning in circles																				
	l) R	Retropulsion - animal walks backwards																				
	m) D	Spatial disorientation -walking or stumbling into ol																				
	n) HB	Head bobbing																				
57.Convulsions	0) No convulsion		0	0	0	0	0	0	0	0	0	0	0	0	0	0	0	0	0	0	0	0
	a-1) C	Clonic																				
	a-2) Cs	Clonic symmetrical																				
	a-3) Rn	Running excitement																				
	a-4) Ch	Champing - clonus of the jaws only																				
	a-5) P	Popcorn																				
	a-6) A	Asphyxia																				
	b-1) T	Tonic																				
	b-(Tf)	Tonic flexation occurs without extension																				
	b-2) Op	Opisthotonus																				
	b-3) Em	Emprosthonus																				
	c-1) Rr	Rock and roll																				
	c-2) Su	Sitting up																				
	c-3) Pr	Praying																				
58.Body Weight (g)			26.7	25.0	25.2	27.8	30.1	27.1	26.4	24.0	27.0	25.9	28.9	27.9	26.9	27.2	29.4	27.7	26.1	27.5	28.5	30.7
