# Supplementary figures and images for: Functional roles of ST8SIA3-mediated sialylation of striatal dopamine D2 and adenosine A2A receptors
Source: Transl Psychiatry. 2019 Aug 27;9:209. doi: 10.1038/s41398-019-0529-z (PMC6712005; doi:10.1038/s41398-019-0529-z)

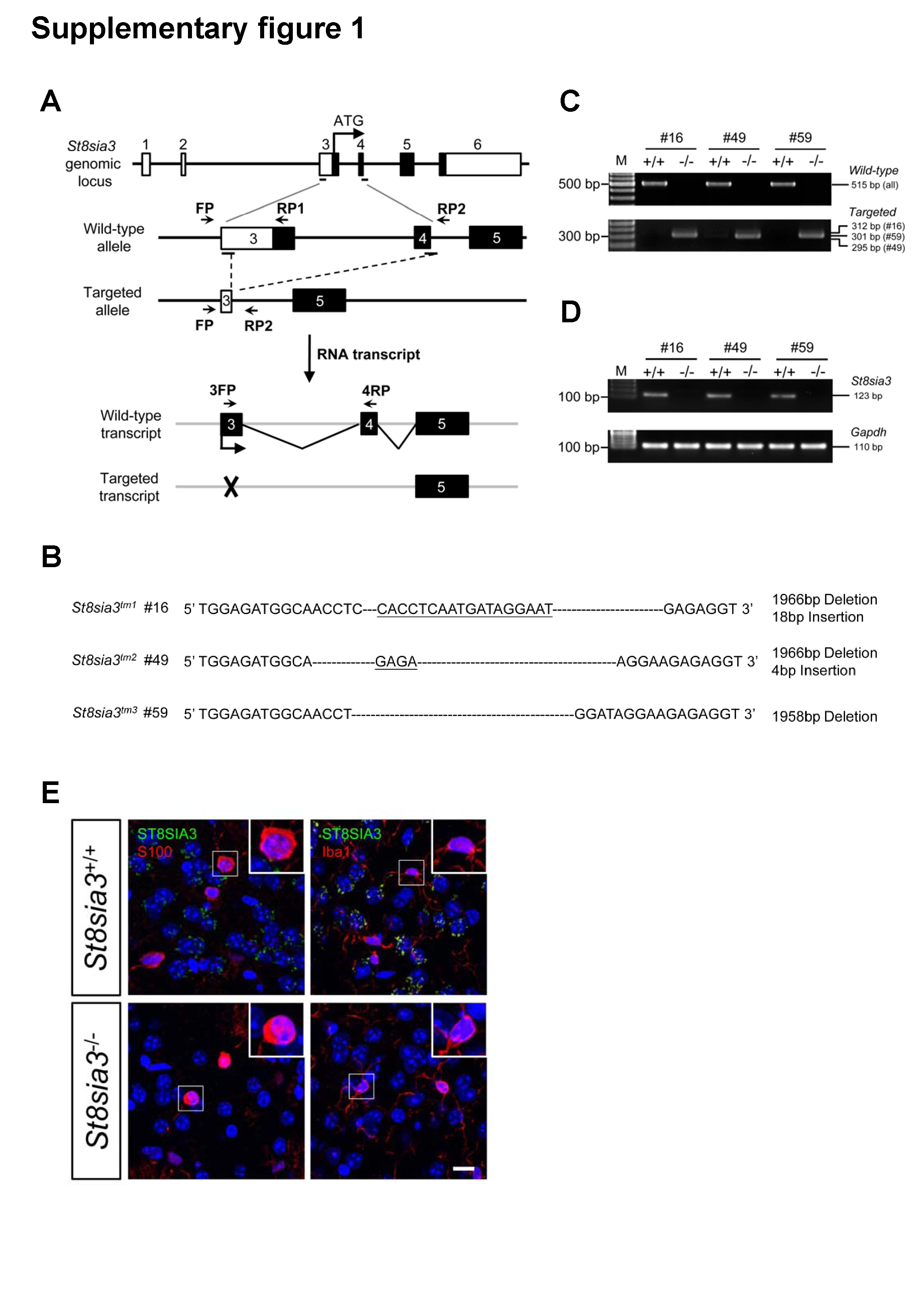

Supplement: Supplementary file 3 — Supplementary Fig. S1 [file 41398_2019_529_MOESM3_ESM.tif]

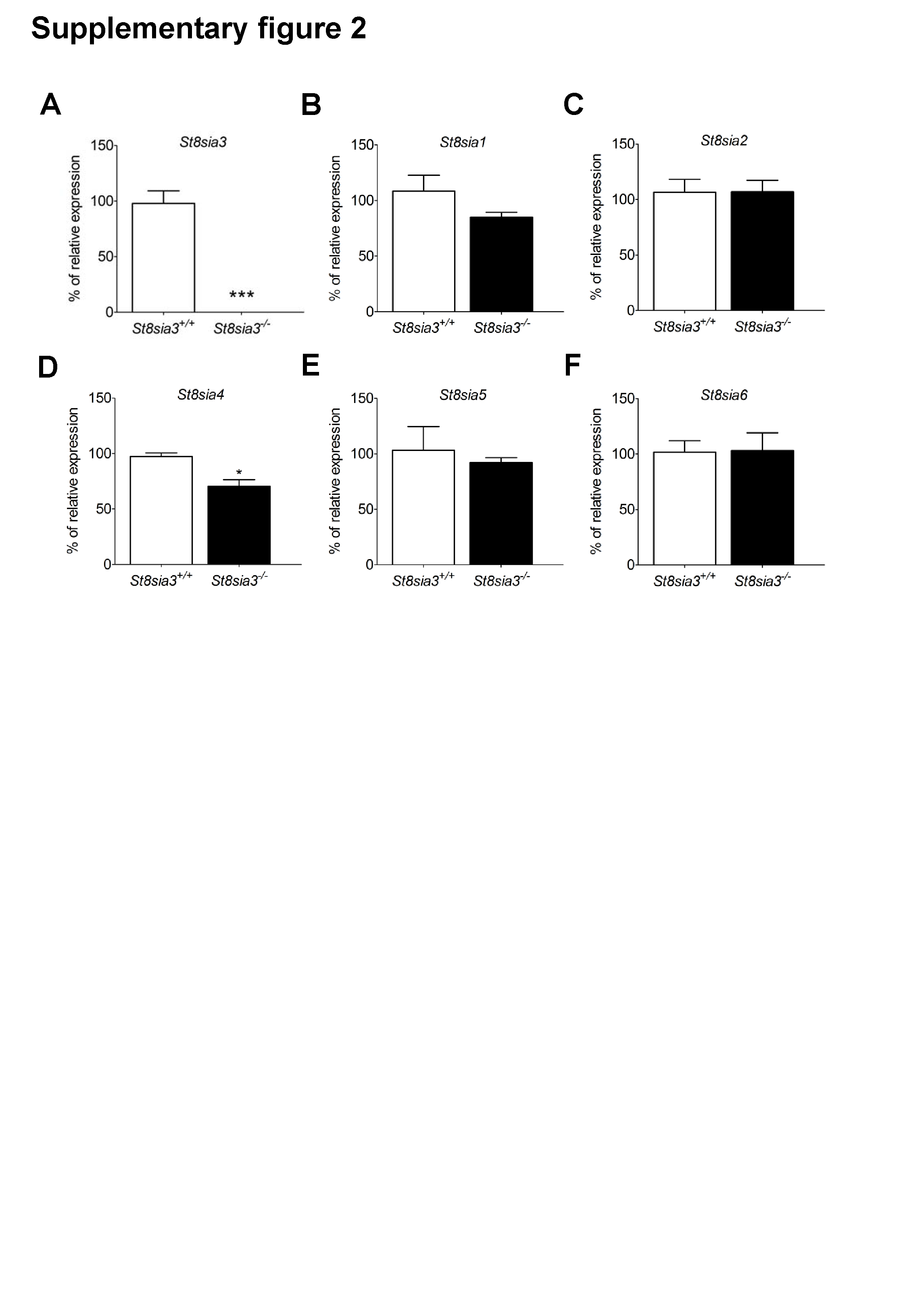

Supplement: Supplementary file 4 — Supplementary Fig. S2 [file 41398_2019_529_MOESM4_ESM.tif]

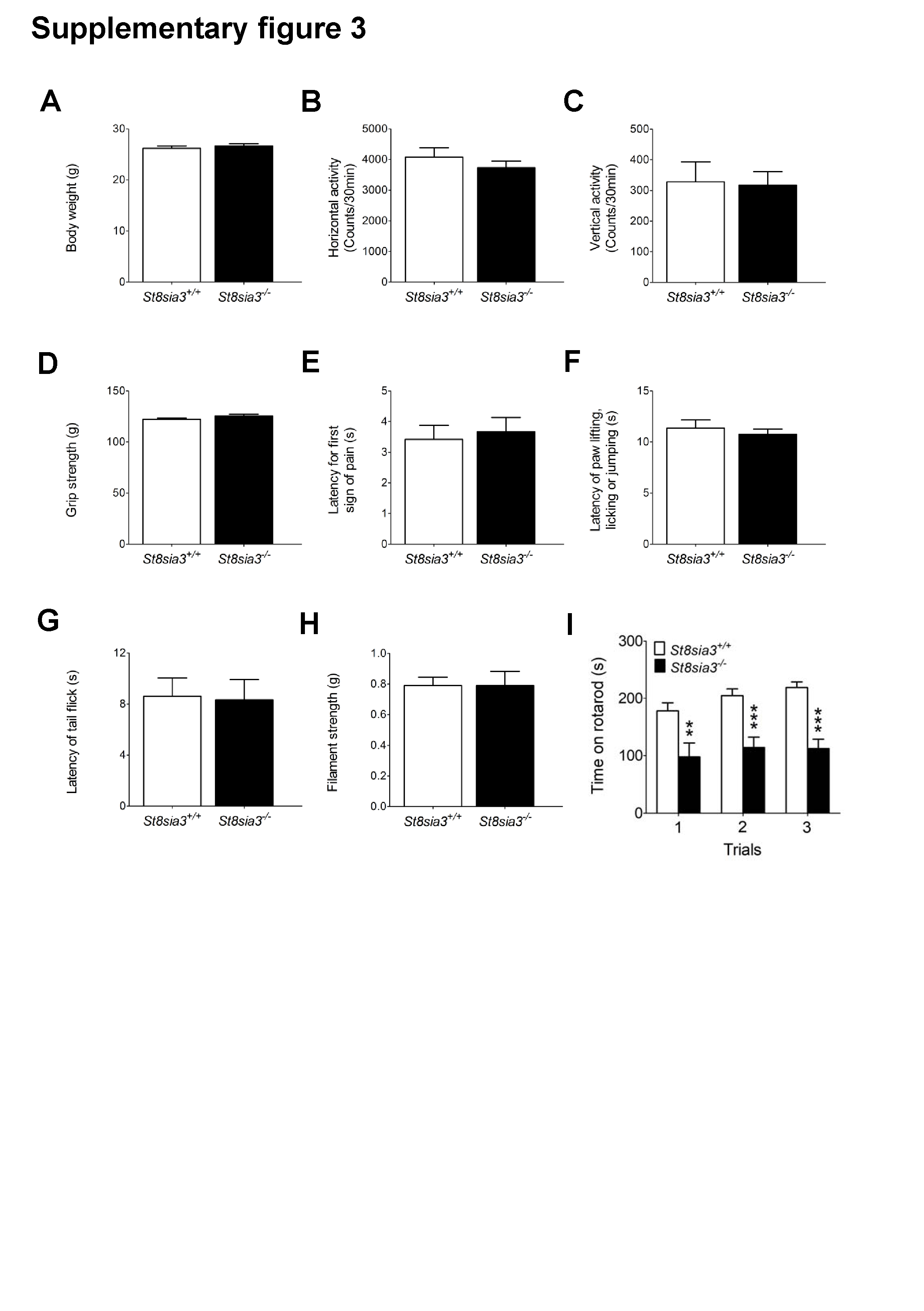

Supplement: Supplementary file 5 — Supplementary Fig. S3 [file 41398_2019_529_MOESM5_ESM.tif]

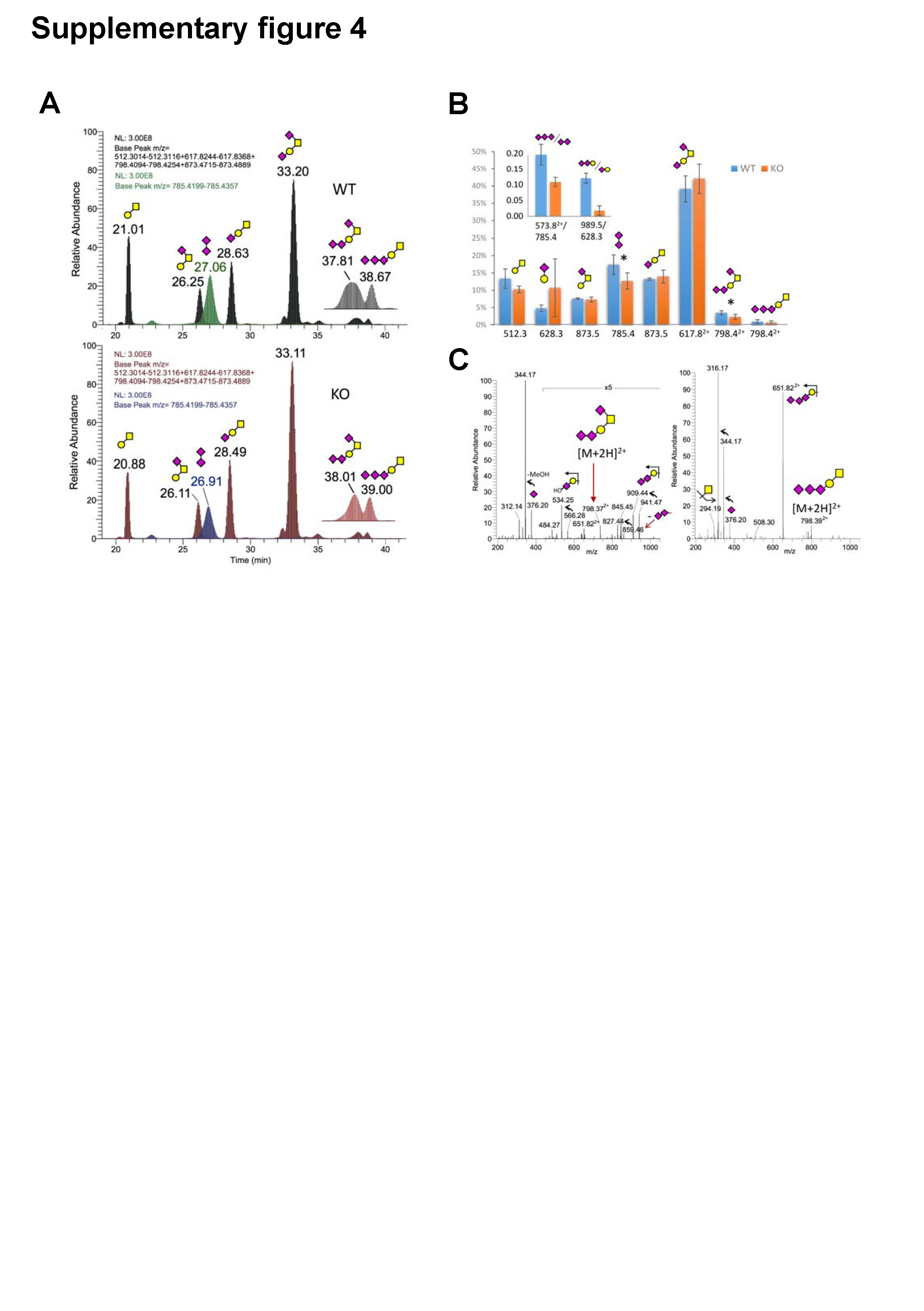

Supplement: Supplementary file 6 — Supplementary Fig. S4 [file 41398_2019_529_MOESM6_ESM.tif]

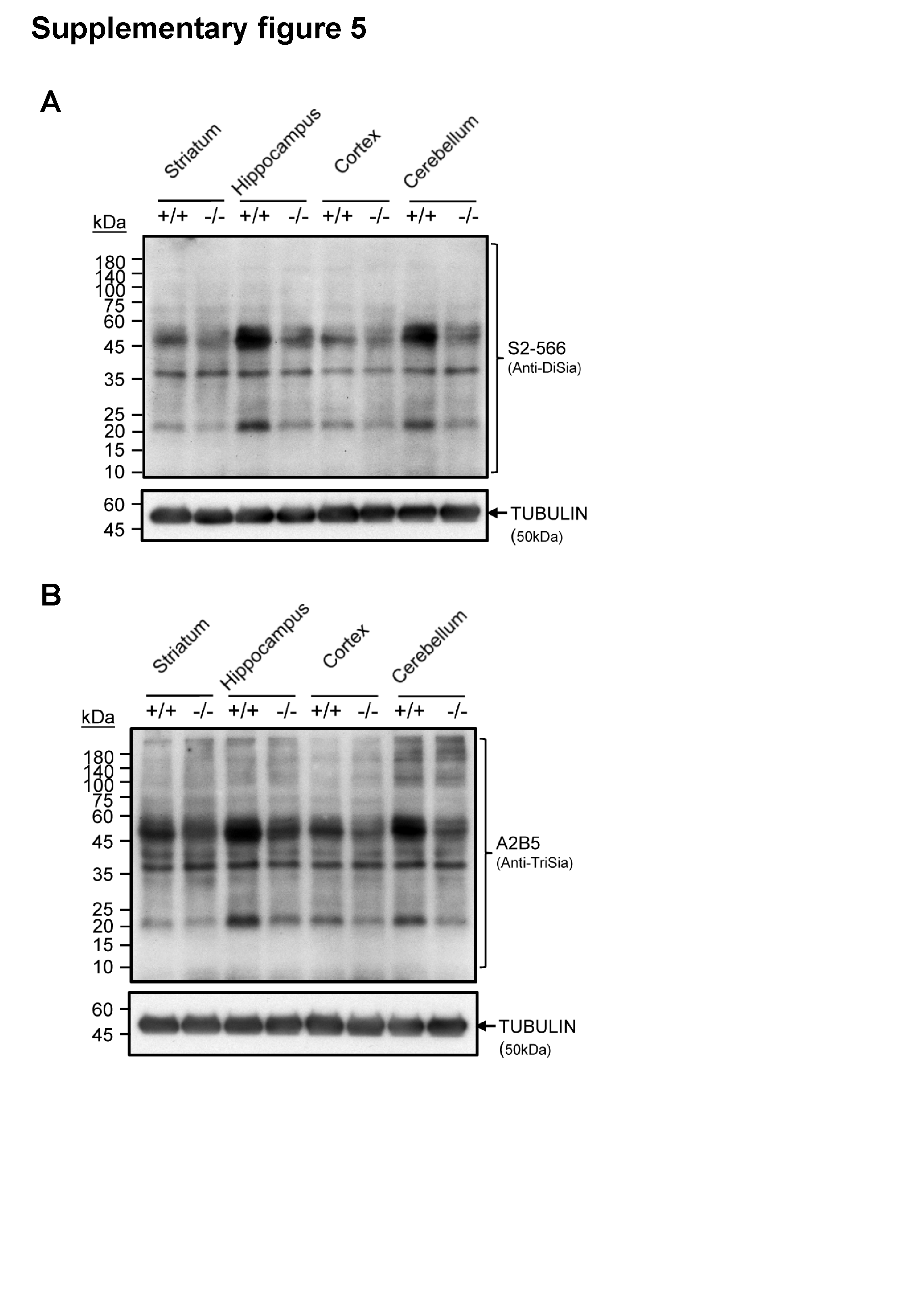

Supplement: Supplementary file 7 — Supplementary Fig. S5 [file 41398_2019_529_MOESM7_ESM.tif]

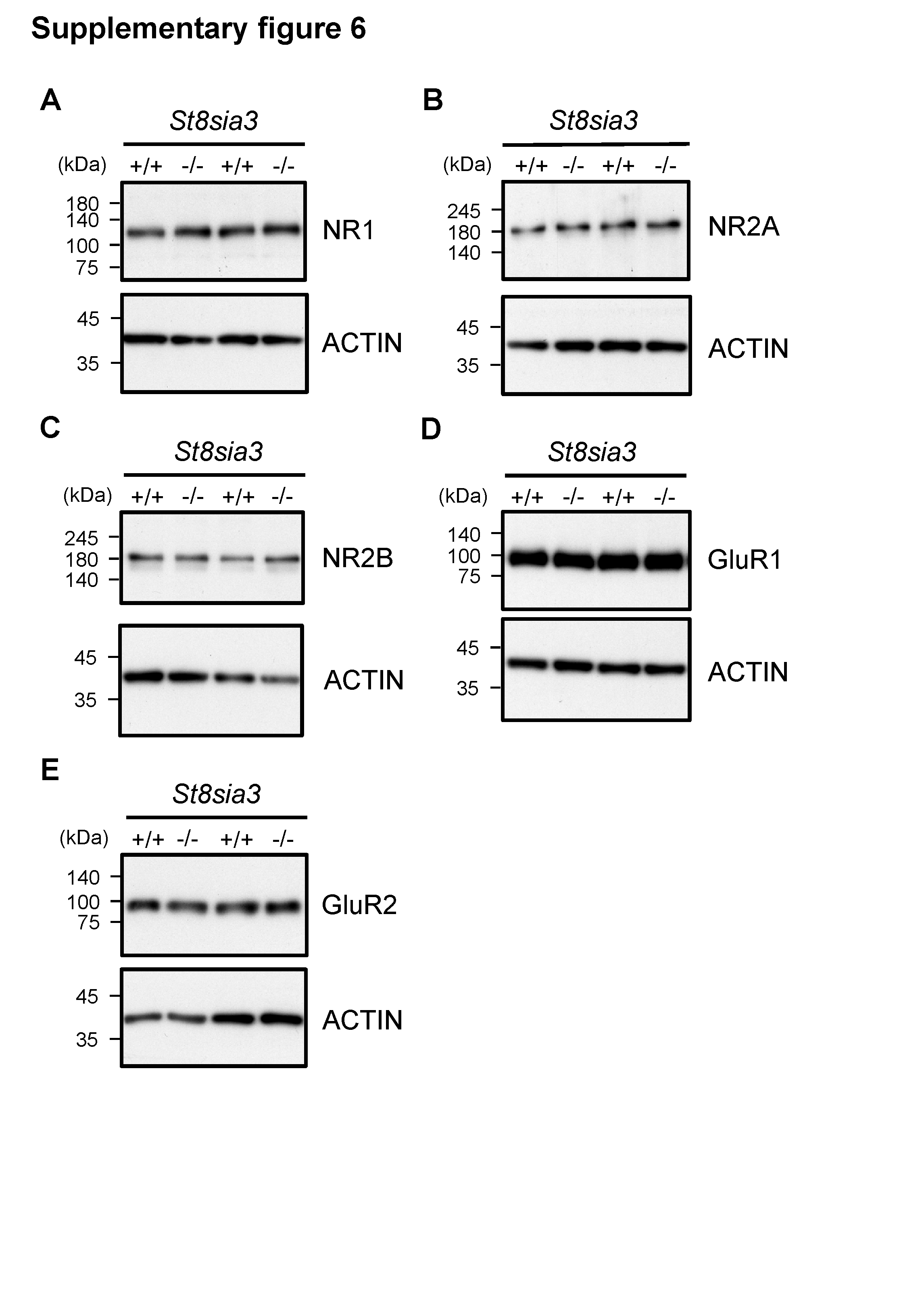

Supplement: Supplementary file 8 — Supplementary Fig. S6 [file 41398_2019_529_MOESM8_ESM.tif]

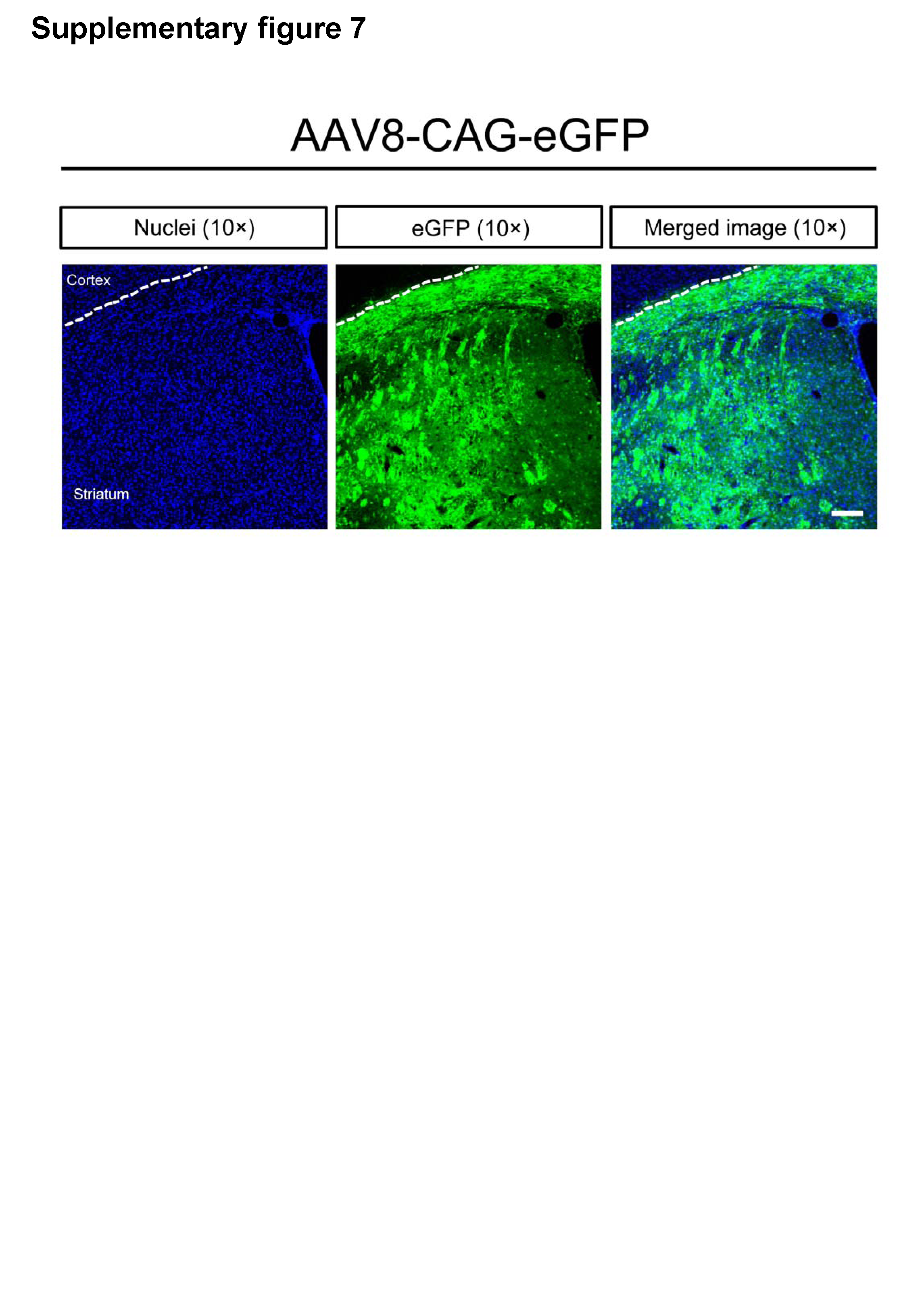

Supplement: Supplementary file 9 — Supplementary Fig. S7 [file 41398_2019_529_MOESM9_ESM.tif]

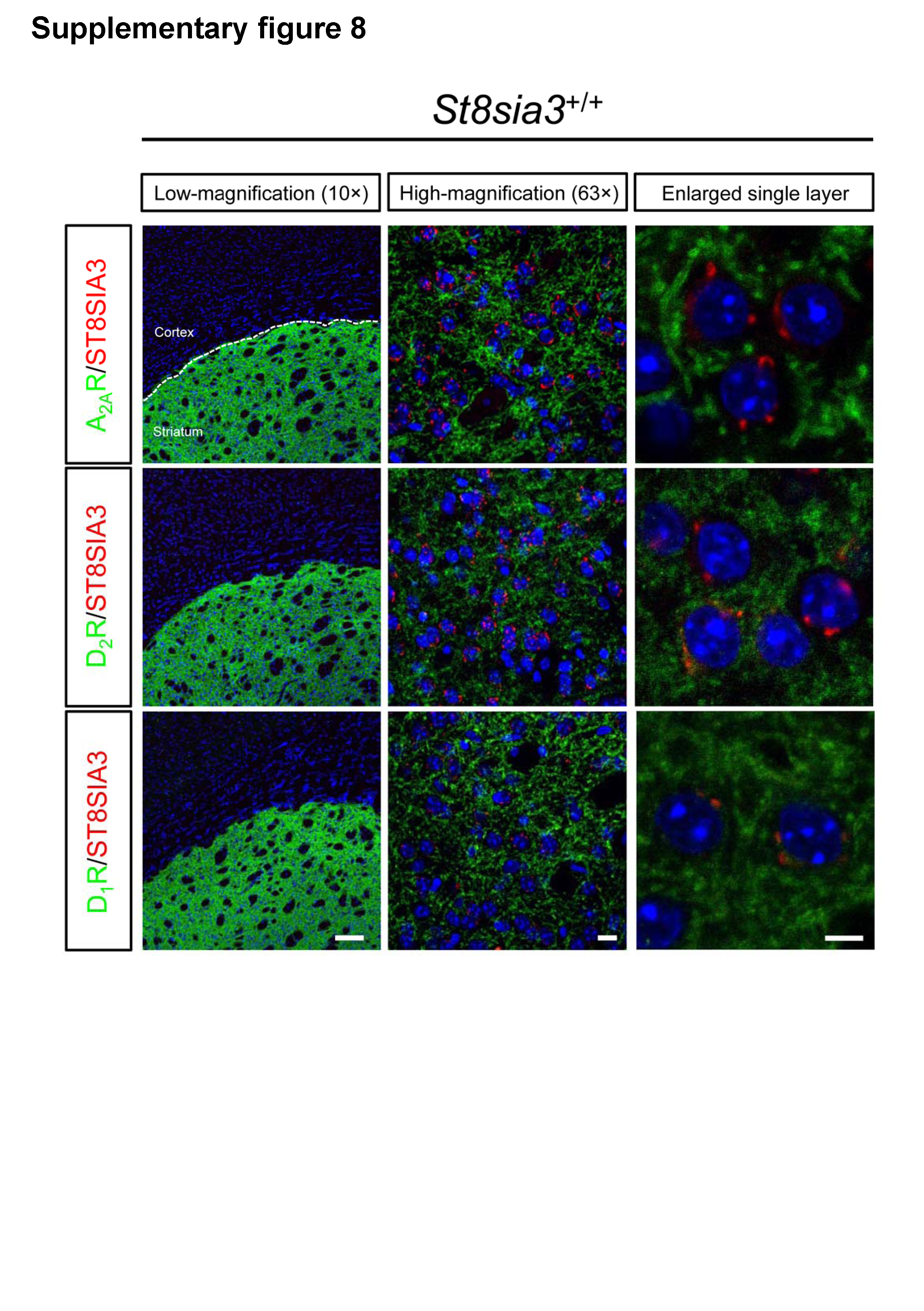

Supplement: Supplementary file 10 — Supplementary Fig. S8 [file 41398_2019_529_MOESM10_ESM.tif]

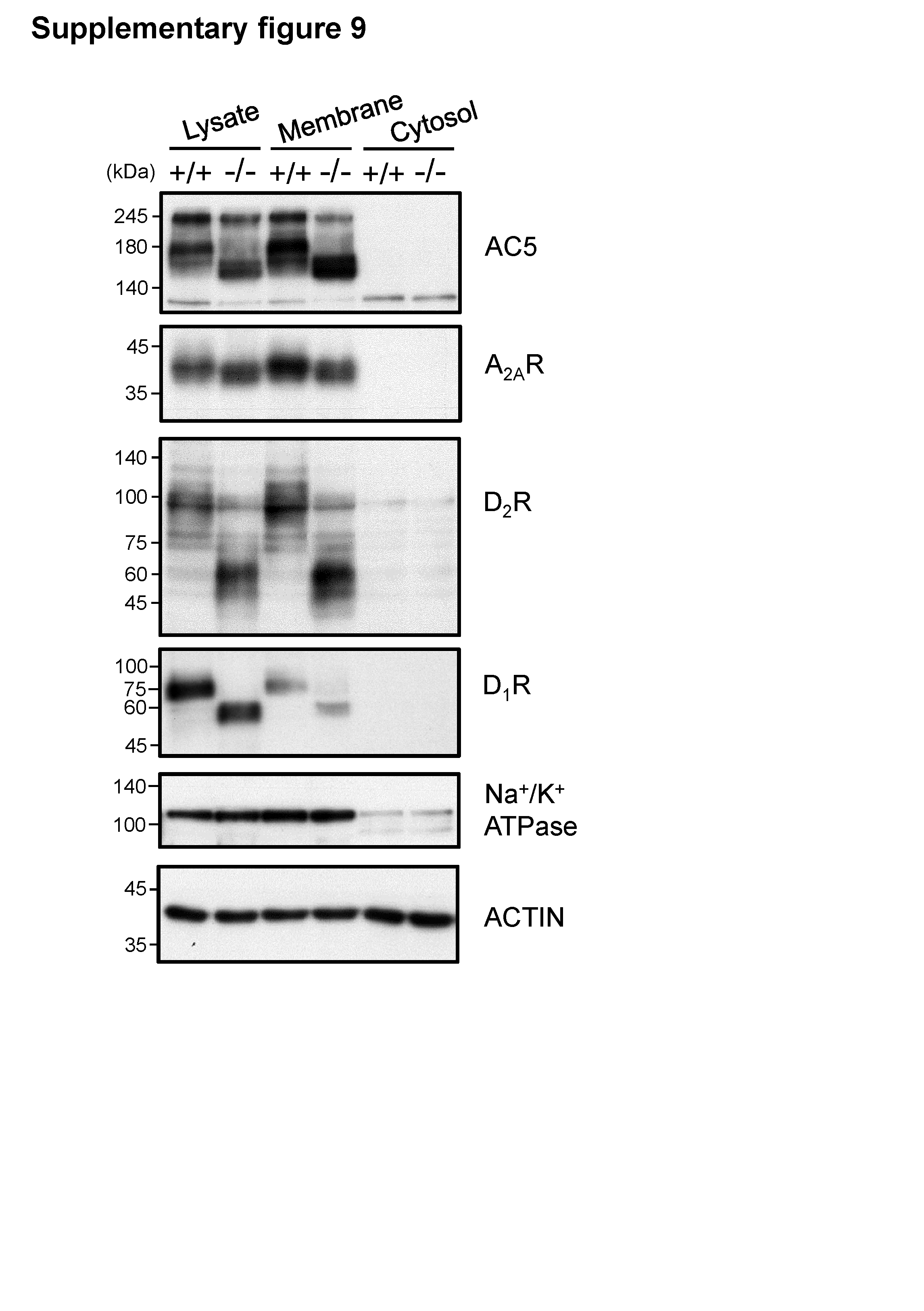

Supplement: Supplementary file 11 — Supplementary Fig. S9 [file 41398_2019_529_MOESM11_ESM.tif]

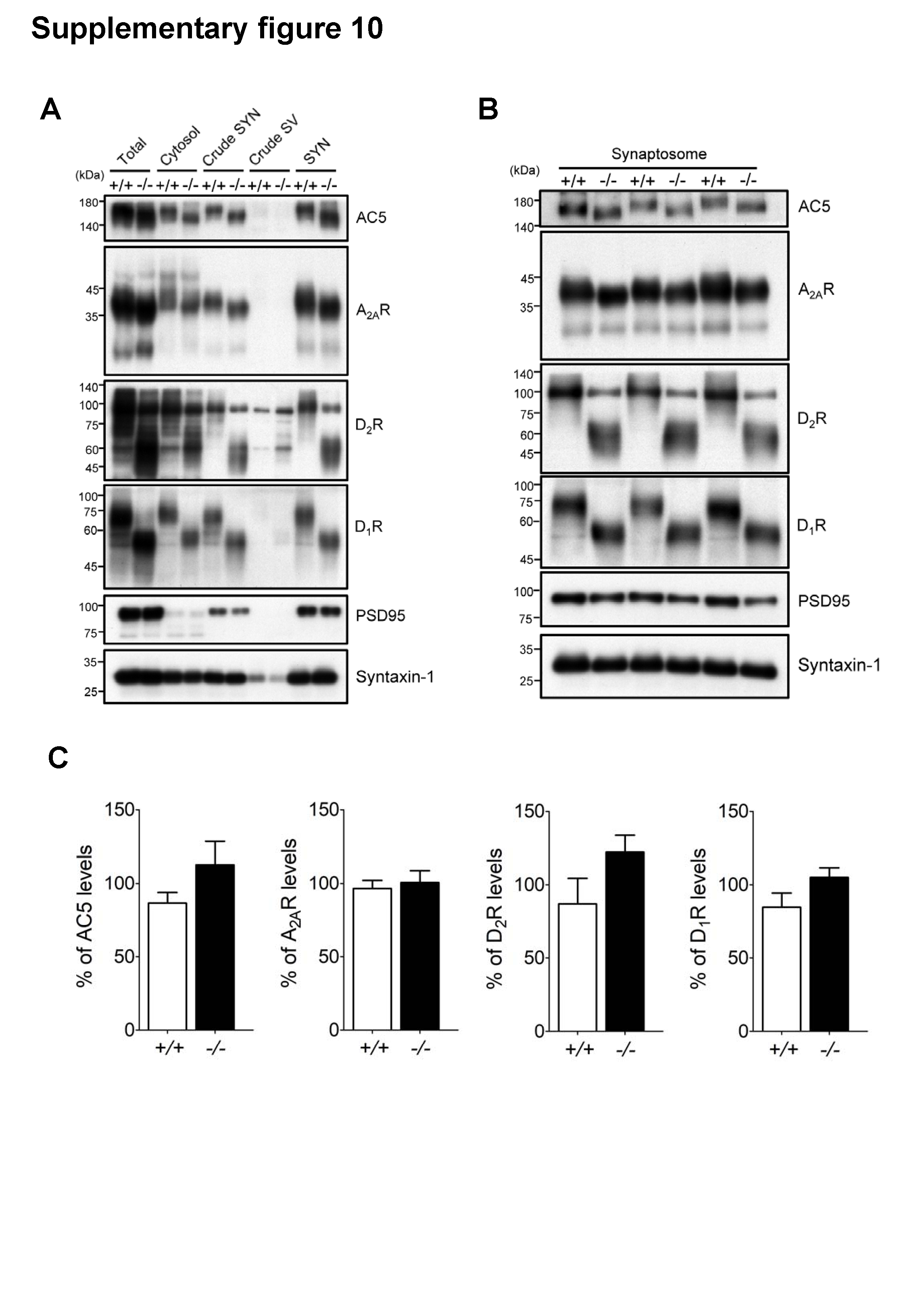

Supplement: Supplementary file 12 — Supplementary Fig. S10 [file 41398_2019_529_MOESM12_ESM.tif]

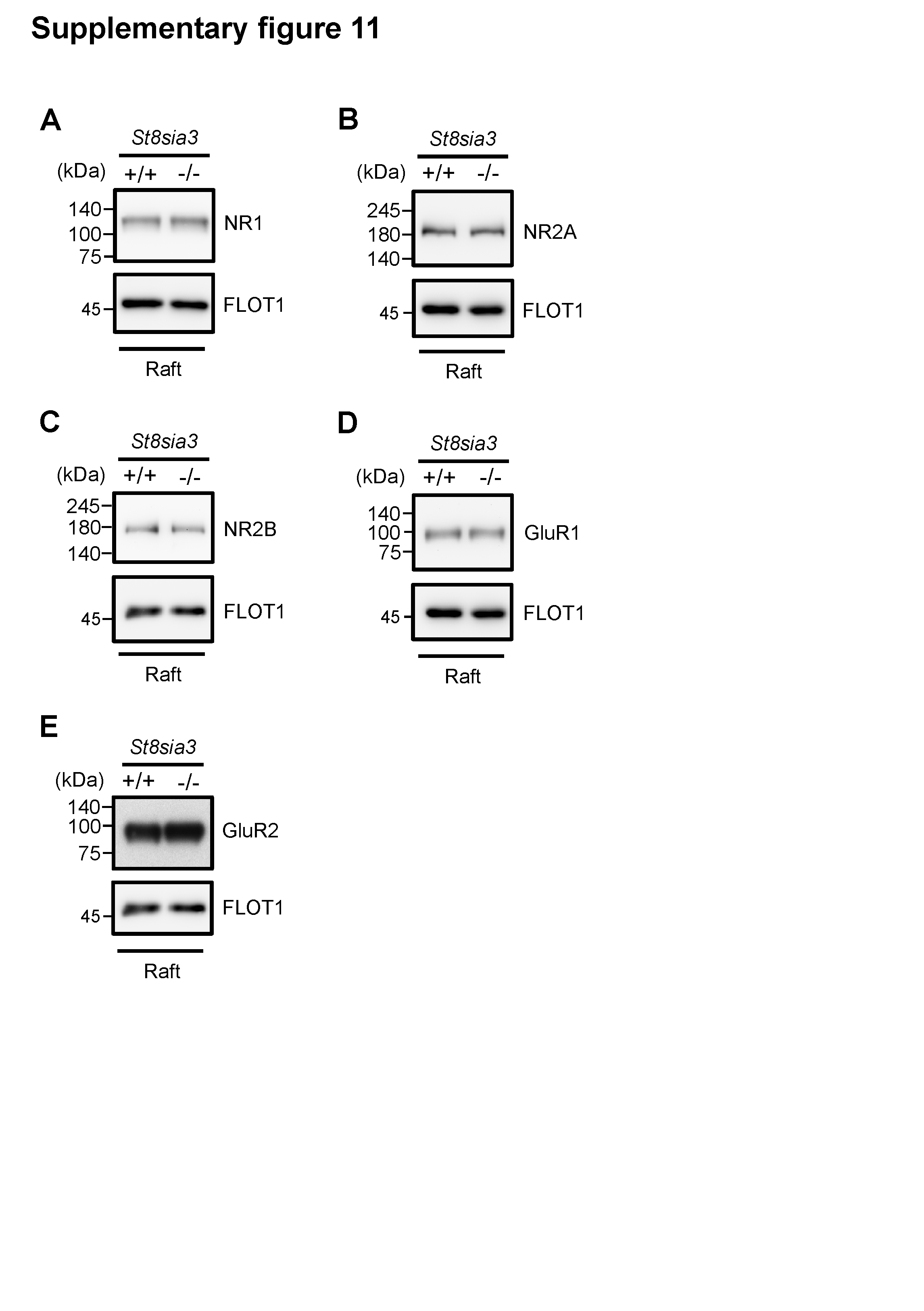

Supplement: Supplementary file 13 — Supplementary Fig. S11 [file 41398_2019_529_MOESM13_ESM.tif]

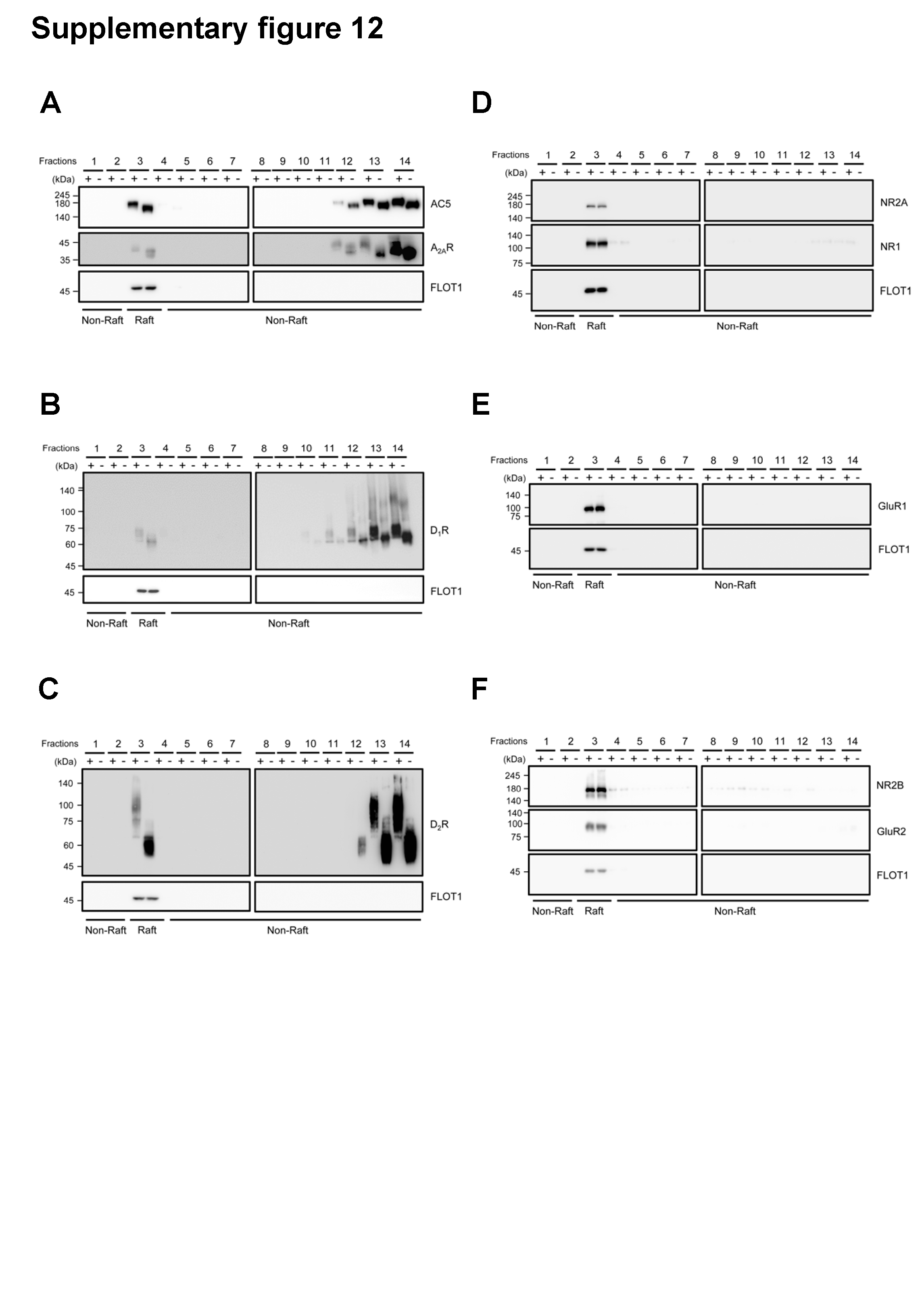

Supplement: Supplementary file 14 — Supplementary Fig. S12 [file 41398_2019_529_MOESM14_ESM.tif]

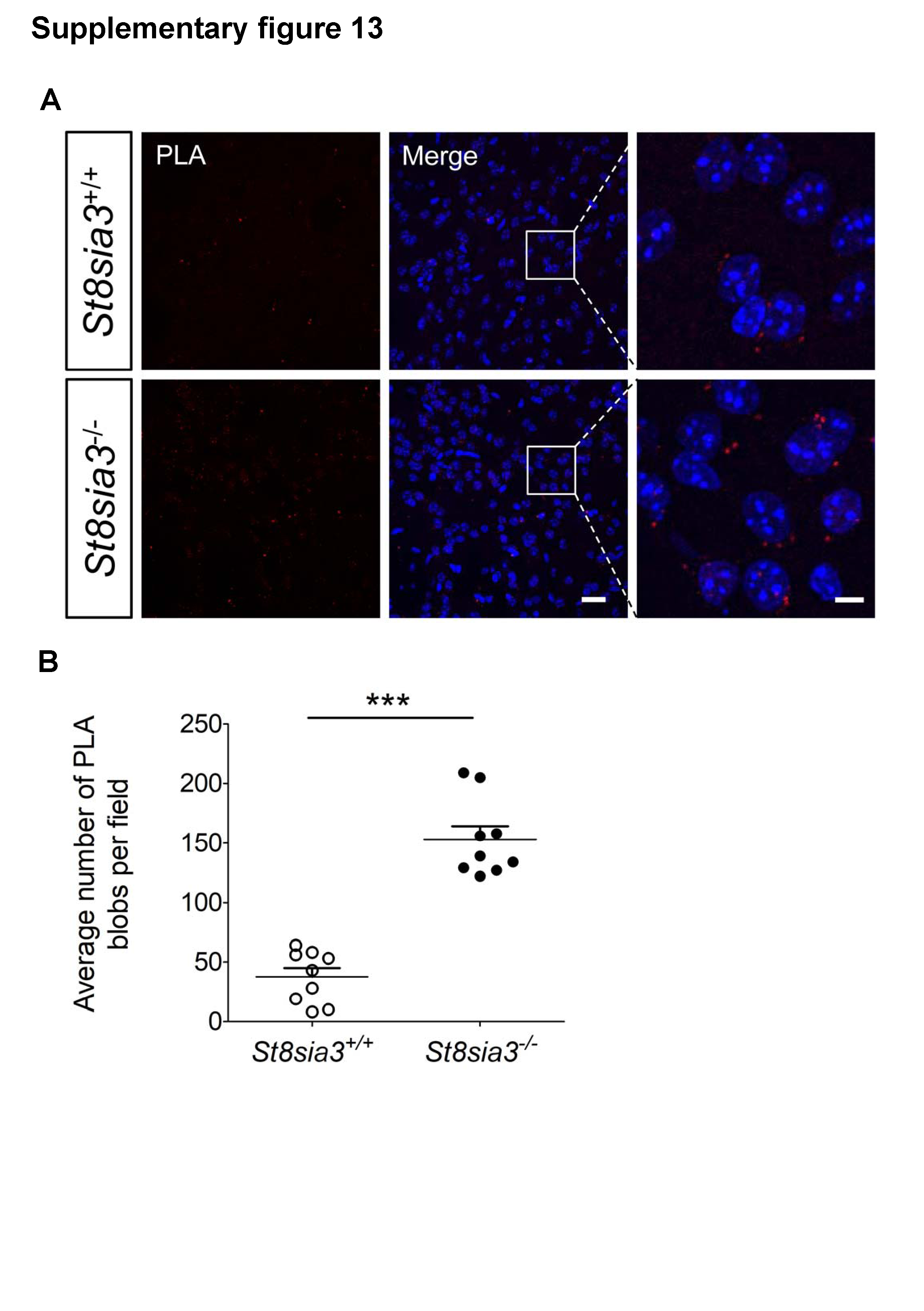

Supplement: Supplementary file 15 — Supplementary Fig. S13 [file 41398_2019_529_MOESM15_ESM.tif]

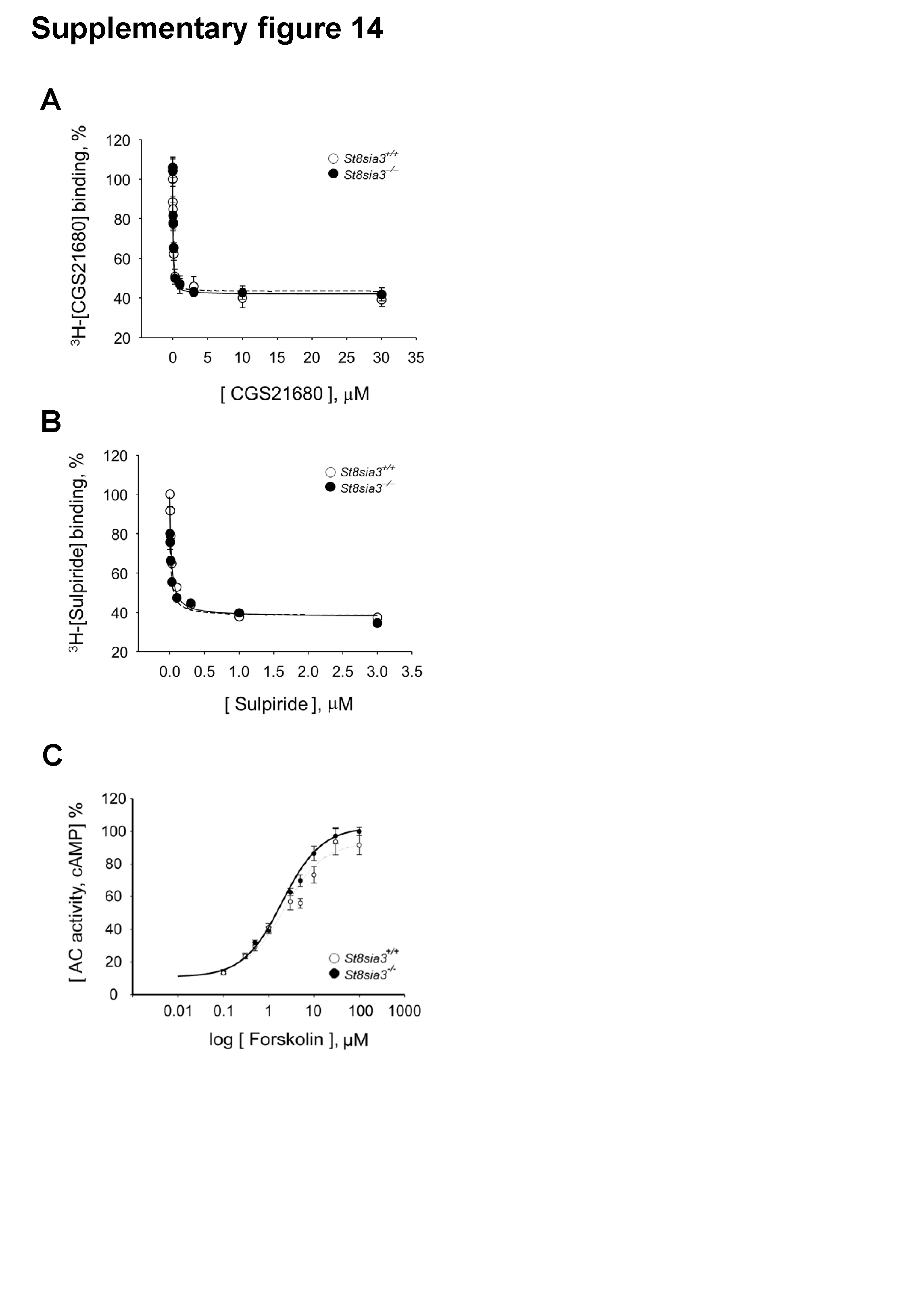

Supplement: Supplementary file 16 — Supplementary Fig. S14 [file 41398_2019_529_MOESM16_ESM.tif]

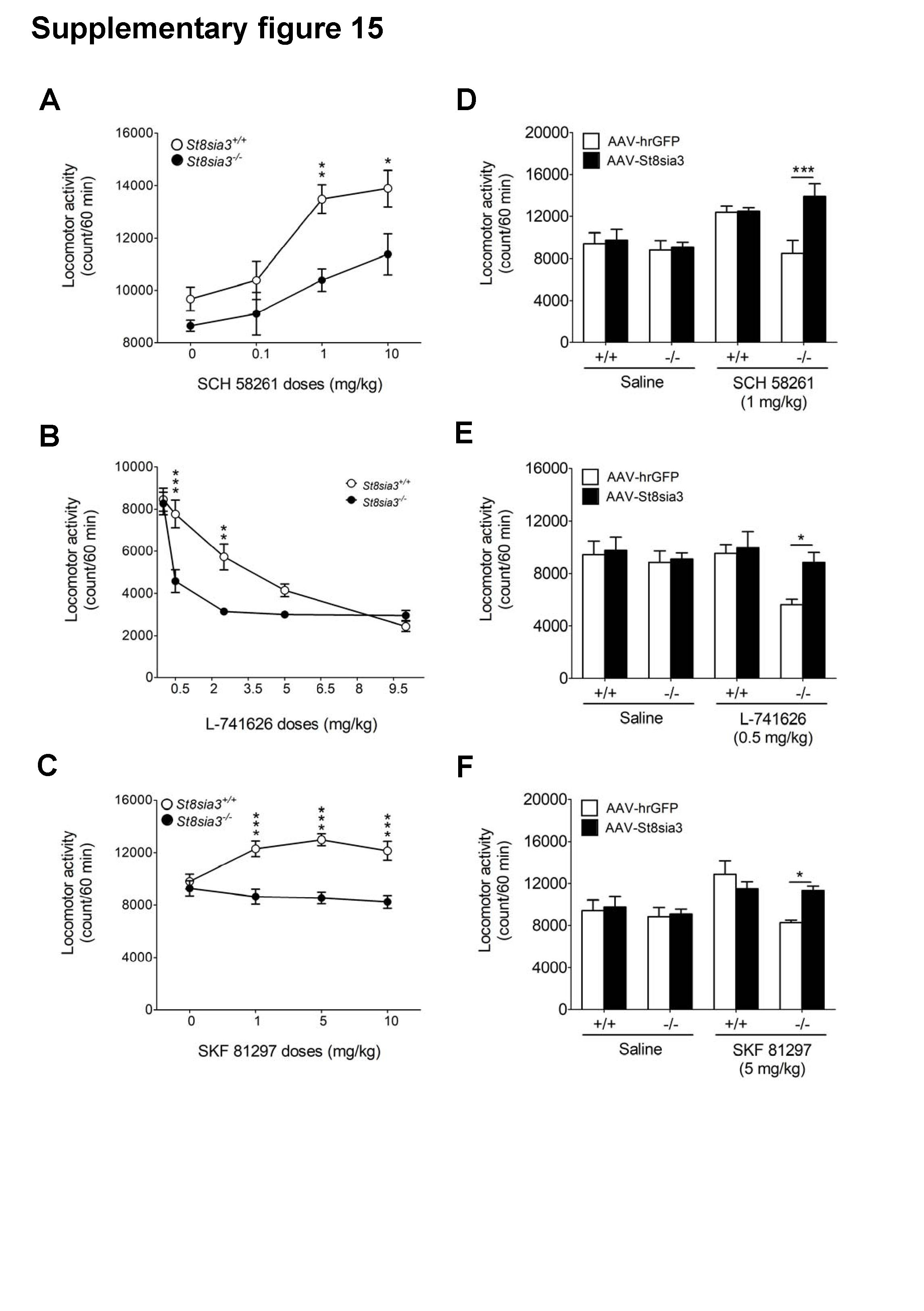

Supplement: Supplementary file 17 — Supplementary Fig. S15 [file 41398_2019_529_MOESM17_ESM.tif]
